# Biogeographical survey of soil microbiomes across sub-Saharan Africa: structure, drivers, and predicted climate-driven changes

**DOI:** 10.1186/s40168-022-01297-w

**Published:** 2022-08-23

**Authors:** DA Cowan, PH Lebre, CER Amon, RW Becker, HI Boga, A Boulangé, TL Chiyaka, T Coetzee, PC de Jager, O Dikinya, F Eckardt, M Greve, MA Harris, DW Hopkins, HB Houngnandan, P Houngnandan, K Jordaan, E Kaimoyo, AK Kambura, G Kamgan-Nkuekam, TP Makhalanyane, G Maggs-Kölling, E Marais, H Mondlane, E Nghalipo, BW Olivier, M Ortiz, LR Pertierra, J-B Ramond, M Seely, I Sithole-Niang, A Valverde, G Varliero, S Vikram, DH Wall, A Zeze

**Affiliations:** 1 Centre for Microbial Ecology and Genomics, Department of Biochemistry, Genetics and Microbiology, University of Pretoria, Pretoria, South Africa; 2 Institut National Polytechnique Houphouet-Boigny, Cote d’Ivoire Yamoussoukro, South Africa; 3 Biodiversity Research Centre, Department of Agriculture and Natural Resources Sciences, Namibia University of Science and Technology, Windhoek, Namibia; 4 Taita Taveta University, Voi, Kenya; 5 Centro de Biotecnologia, Universidade Eduardo Mondlane, Maputo, Mozambique; 6 UMR InterTryp, CIRAD-IRD, 34398 Montpellier, France; 7 Department of Biotechnology and Biochemistry, University of Zimbabwe, Harare, Zimbabwe; 8 Department of Plant and Soil Sciences, University of Pretoria, Pretoria, South Africa; 9 Department of Environmental Science, University of Botswana, Gaborone, Botswana; 10 Department of Geography, University of Cape Town, Cape Town, South Africa; 11 Scotland’s Rural College, Edinburgh, EH9 3JG UK; 12 Université Nationale d’Agriculture, Porto-Novo, Benin (Laboratoire de Microbiologie Des Sols Et d’Ecologie Microbienne), Porto-Novo, Benin; 13 Departamento de Genética Molecular Y Microbiología, Facultad de Ciencias Biológicas, Pontificia Universidad Católica de Chile, Santiago, Chile; 14 University of Zambia, Lusaka, Zambia; 15 Department of Biochemistry, Genetics and Microbiology, University of Pretoria, Pretoria, South Africa; 16 Gobabeb-Namib Research Institute, Walvis Bay, Namibia; 17 Department of Biological Sciences, Clemson University, Clemson, SC USA; 18 Department of Biology, Colorado State University, Fort Collins, USA

**Keywords:** Sub-Saharan Africa, Soil microbiome, Microbial biodiversity, Climate change, Ecosystem predictions

## Abstract

**Background:**

Top-soil microbiomes make a vital contribution to the Earth’s ecology and harbor an extraordinarily high biodiversity. They are also key players in many ecosystem services, particularly in arid regions of the globe such as the African continent. While several recent studies have documented patterns in global soil microbial ecology, these are largely biased towards widely studied regions and rely on models to interpolate the microbial diversity of other regions where there is low data coverage. This is the case for sub-Saharan Africa, where the number of regional microbial studies is very low in comparison to other continents.

**Results:**

The aim of this study was to conduct an extensive biogeographical survey of sub-Saharan Africa’s top-soil microbiomes, with a specific focus on investigating the environmental drivers of microbial ecology across the region. In this study, we sampled 810 sample sites across 9 sub-Saharan African countries and used taxonomic barcoding to profile the microbial ecology of these regions. Our results showed that the sub-Saharan nations included in the study harbor qualitatively distinguishable soil microbiomes. In addition, using soil chemistry and climatic data extracted from the same sites, we demonstrated that the top-soil microbiome is shaped by a broad range of environmental factors, most notably pH, precipitation, and temperature. Through the use of structural equation modeling, we also developed a model to predict how soil microbial biodiversity in sub-Saharan Africa might be affected by future climate change scenarios. This model predicted that the soil microbial biodiversity of countries such as Kenya will be negatively affected by increased temperatures and decreased precipitation, while the fungal biodiversity of Benin will benefit from the increase in annual precipitation.

**Conclusion:**

This study represents the most extensive biogeographical survey of sub-Saharan top-soil microbiomes to date. Importantly, this study has allowed us to identify countries in sub-Saharan Africa that might be particularly vulnerable to losses in soil microbial ecology and productivity due to climate change. Considering the reliance of many economies in the region on rain-fed agriculture, this study provides crucial information to support conservation efforts in the countries that will be most heavily impacted by climate change.

Video Abstract

**Supplementary Information:**

The online version contains supplementary material available at 10.1186/s40168-022-01297-w.

## Introduction

The top-soil microbiome has been recognized for more than a century as a crucial part of the Earth’s ecology [1]. Not only are soil microorganisms ubiquitous across most terrestrial environments [2, 3], but they also play important roles in the maintenance of soil fertility through nutrient cycling as well as carbon and nitrogen sequestration [4–6]. Major studies, led by the Earth Microbiome Project [7], have attempted to map both the microbial diversity and functional capacity of soil microbial communities across the globe by using a combination of sequence-based approaches, including taxonomic marker gene (e.g., 16S rRNA gene and ITS) DNA-barcoding approaches and more in-depth next-generation sequencing of total soil DNA (see Fierer (2017) for a comprehensive review [8]). These and many other more localized studies [9–13] have shown that a small percentage of microbial taxa dominate global soil microbial communities, with phyla such as the bacterial Acidobacteriota and Proteobacteria, the archaeal Crenarchaeota, and the fungal Ascomycota [14–17] being commonly found within soil microbiomes. However, contrary to the popular Baas-Becking hypothesis which states that “everything is everywhere, but the environment selects” [18], microecological surveys have shown that different biomes harbor distinct microbial communities that are shaped by both deterministic processes such as environment selection, as well as more stochastic events such as dispersal limitation [19–21].

The heterogeneity of soil conditions, even at the centimeter-scale [22], results in similarly heterogeneous microbial patterns within and between soils that defy the concept of a “typical” soil microbiome across any specific environment [8]. In addition to this spatial variability, the plurality of sampling and analysis methods employed by the microbiology research community have made it difficult to reach a holistic model of the interactions between microbial communities and their environments. For instance, some studies on the global soil microbiome have identified pH as the best predictor of microbial variability [23, 24], while others have highlighted carbon availability [25], as well as salinity [26], as strong drivers of microbial community structure. The differences between these results might be due to different spatial scales, differences in the range of soil physical, and chemical conditions, as well as methodological differences.

More recently, much attention has been focused on the shifts in the global soil microbiome as a result of climate change [27–29]. Human-driven climate change has already resulted in drastic changes in the microecology of some of Earth’s most climate-sensitive biomes, such as polar deserts and semi-arid arable lands [30]. The recent rise in global temperatures, caused by the accumulation of greenhouse gasses, has driven the accelerated thaw of permafrost soils in the Arctic, which results in a rapid shift in the diversity and functional profile of permafrost microbial communities [31, 32]. This shift has been characterized by the increased decomposition of soluble organic carbon and increased production of the greenhouse gases CO_2_ and CH_4_, which generate a feedback loop for stimulated global warming [33]. Similarly, increased drought as a consequence of altered precipitation patterns is predicted to drive the accelerated desertification of semi-arid and arid regions [34] and result in the decreased productivity in the soil microbiome [35]. Furthermore, drought is thought to have a long-lasting impact on soil microbial communities due to shifts in vegetation to more drought-tolerant plant species, which drive significant changes in the composition of root-associated microbial communities [36].

Sub-Saharan Africa is particularly vulnerable to climate change. There is evidence that the African continent is warming at a faster rate than the rest of the globe [37] and that drought/flood disaster events, which already constitute 25% of disasters on the continent [38], are projected to increase due to effects of climate change [39]. As a result, many sub-Saharan African countries are expected to face increased water stress and scarcity by 2025 [38, 40], putting a severe strain on the largely rain-fed agricultural economies in these regions and consequently on human livelihoods [41]. Additionally, increased drought periods combined with soil erosion from anthropogenic land misuse will further increase the rate of land desertification [42, 43], which has already been shown to have a detrimental impact on both macro- and micro-ecology of affected areas [44, 45]. Considering the importance of the top-soil microbiome in providing a broad range of ecosystem services [46–48] and maintaining soil health [27, 49, 50], the loss of soil biodiversity and function associated with desertification will likely exacerbate the challenges to ecology and soil productivity already felt in sub-Saharan Africa. However, despite the relevance of the soil microbiome to the ecology and soil health of sub-Saharan Africa, to date, there has been no comprehensive survey of the top-soil microbiome in this region [51]. This gap in the knowledge of global micro-ecology has led us to perform an extensive microbial community survey of the soils across sub-Saharan Africa, with three specific aims: (1) to document the microbial biodiversity across sub-Saharan Africa, (2) to determine how the environment affects soil community biodiversity and structure in this region, and (3) to infer the potential impacts of environmental change on the biodiversity and composition of the sub-Saharan Africa top-soil microbiome. We hypothesize that the soil microbial ecology will be significantly distinct across the different regions of the sub-continent and will be shaped by a combination of soil chemistry variables and climate. In addition, we hypothesize that the effects of climate change will have a measurable impact on the diversity and composition of sub-Saharan soil microbiomes. This study also provides a much-needed baseline for future analyses aimed at assessing the qualitative and quantitative impacts of climate change on soil microbiomes.

## Results and discussion

### African nations have distinct soil physicochemical properties

**Fig. 1:**
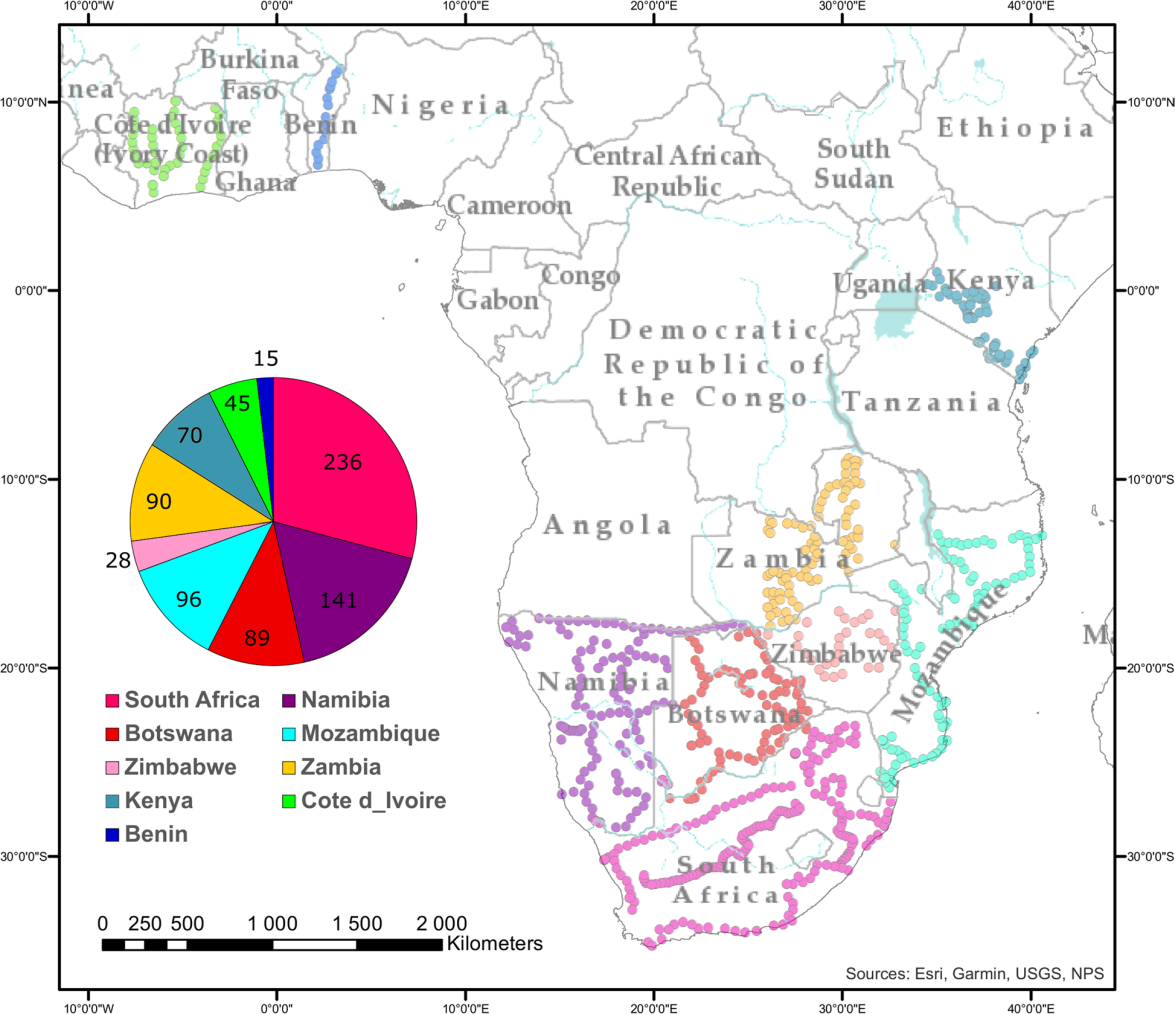
Maps of the sub-Saharan African showing the sites from which soils were extracted for this study and pie chart representing the distribution of samples according to the sampled countries. Sampled sites in the map are represented by dots which are colored according to the country of origin

**Fig. 2:**
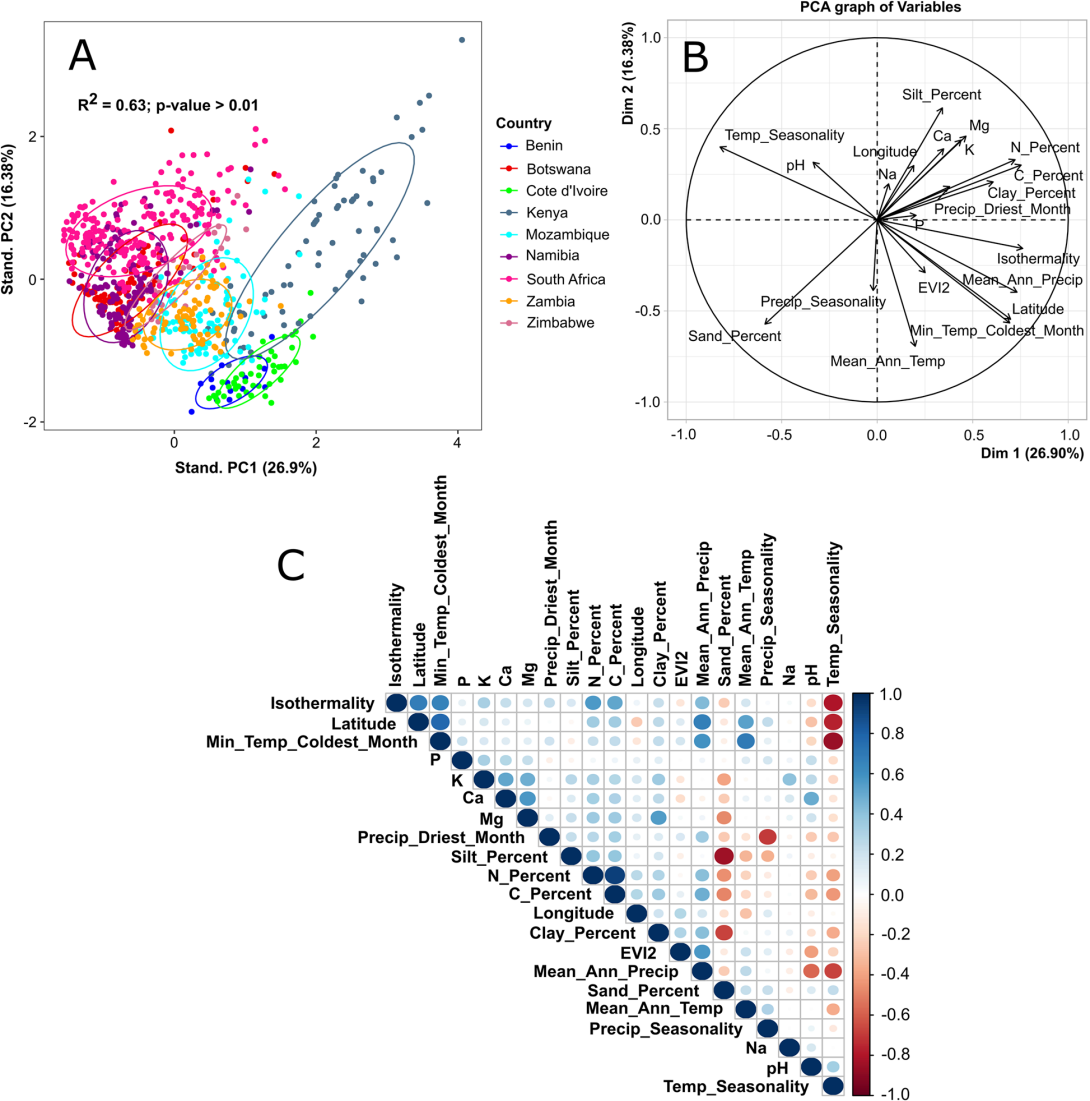
**A**, **B** Principal component analysis (PCA) biplot of all soil samples according to their chemistry and climatic properties. The influence of each variable on sample distribution is represented by the arrows radiating from the center of the PCA plot. The sample clusters corresponding to the different countries are highlighted within the ellipses of the same color. **C** Pearson correlation between soil chemistry and climatic variables. Positive and negative correlations are displayed in blue and red, respectively, while the size and intensity of matrix circles is proportional to correlation coefficient between variables. The description and units for each variable code can be found in Table S8

In this study, we obtained surface soil samples from an extensive area across sub-Saharan Africa, spanning 9 countries and multiple biomes. Of the total of 810 soil samples collected, the majority of samples were from South Africa (236), followed by Namibia (141) and Botswana (89) (Fig. 1, Table S1). In addition to the countries of origin, sample sites were assigned to biomes, based on their vegetation land cover (LC) (Figure S1, Table S1). None of the sampled countries was dominated by a single biome, but Namibia and Botswana contained the highest percentage of bare and poorly vegetated soils, while South Africa and Kenya showed the highest diversity of sampled biomes. The diversity of biomes in Kenya is particularly striking, considering that the sample size and area covered in this country are considerably smaller than in South Africa, where one would expect to have a higher diversity of biomes [52, 53]. Soil chemistry of the samples and climatic data taken from the sampling sites revealed that countries could be significantly distinguished (*p*-value < 0.01, *R*^2^ = 0.63) based on soil nutrient composition, soil pH, vegetation cover, and precipitation (Fig. 2A, Figure S2). These results reflect the distribution of distinct biomes across sub-Saharan African countries, which are themselves defined by differences in nutrient composition, pH, and climate.

Many of the measured soil chemistry and climatic variables were found to be correlated and therefore could be considered as co-dependent (Fig. 2B, C). For instance, mean annual precipitation (MAP) was negatively correlated with pH and positively correlated with the soil vegetation index (EVI2) as well as both organic carbon and nitrogen content. As expected, MAP was also strongly correlated with the level of vegetation, with more vegetated biomes exhibiting higher levels of MAP. These results are consistent with the well-documented role of precipitation as an important driver of plant growth and soil fertility [54–56]. MAP also decreased with decreasing latitude, a trend that has been previously documented as an effect of climate change [57]. In turn, the vegetation index was positively correlated with soil nutrients, which is expected as EVI2 is often used as a proxy for plant productivity [58].

Another relevant relationship observed across sub-Saharan African countries was the inverse correlation between temperature seasonality and MAP, soil vegetation, and soil nutrients. Temperature seasonality is defined as the amount of temperature variation across the year [59], and extreme temperature seasonality has been shown to be one of the more debilitating effects of climate change, leading to severe decreases in ecosystem biodiversity and functionality [60, 61]. Temperature seasonality was also found to be more severe at lower latitudes, again highlighting the high vulnerability of lower latitude sub-Saharan African countries to the effects of climate change.

### Ecologically important phyla are ubiquitous in sub-Saharan African soils

**Fig. 3:**
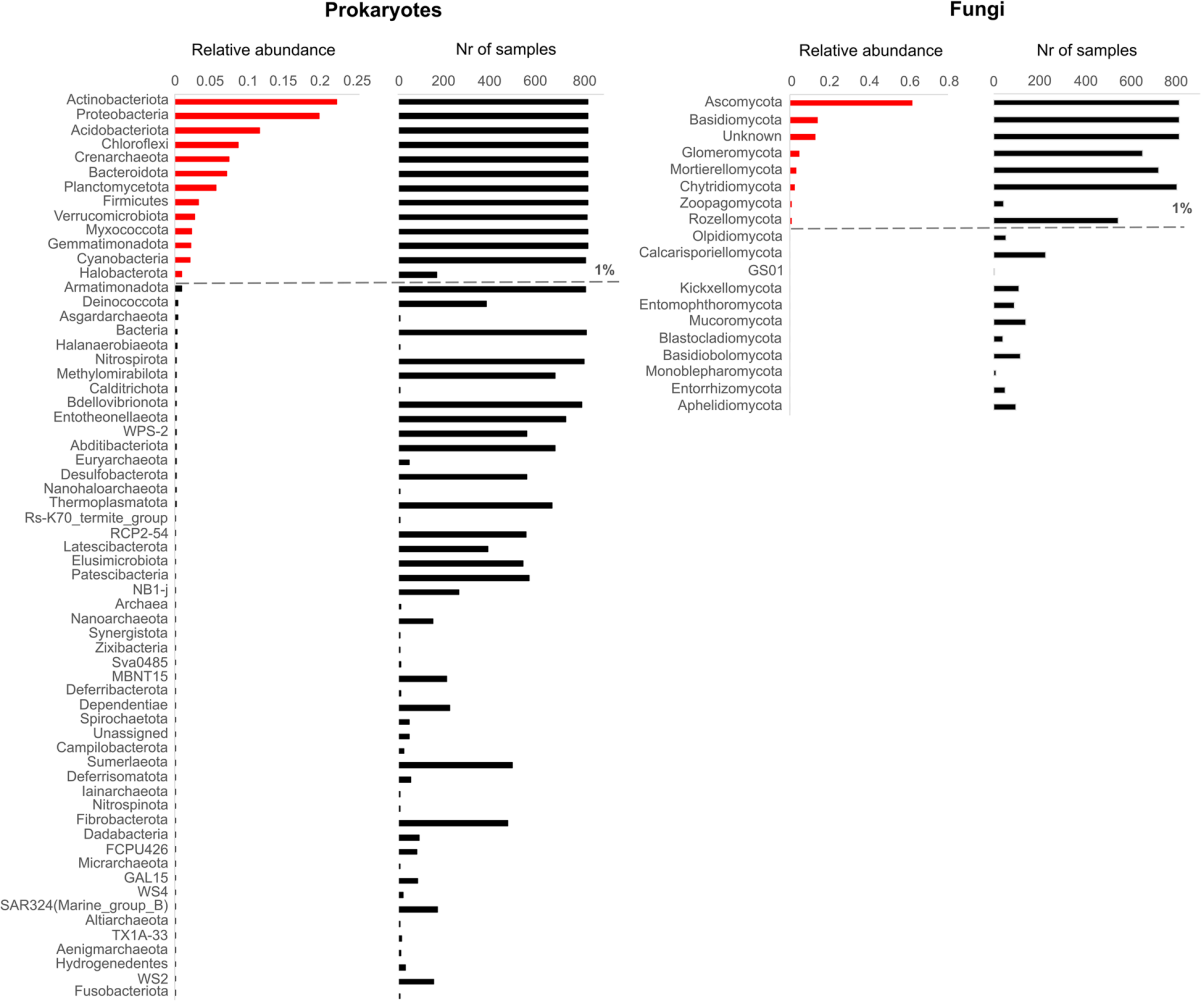
Mean relative abundances (expressed a fraction of total abundance) of prokaryotic and fungal phyla across all sub-Saharan African soil samples, together with the number of samples in which they were identified. Dominant phyla, defined as phyla with more than 1% mean relative abundance, are highlighted in red above dashed gray lines, which represent the threshold between dominant and rare taxa

Several large-scale microbial surveys have documented the dominance of a relatively small number of prokaryotic phyla across global soil microbiomes [8, 23, 26]. This study has revealed a broadly similar pattern for sub-Saharan African soils, with only 13 of a total of 63 prokaryotic phyla representing the dominant fraction (i.e., phyla represented by more than 1% mean relative abundance across samples) of the soil microbiome (Fig. 3). Twelve of these were also distributed across the majority (> 98%) of samples, further highlighting their classification as ubiquitous taxa. The most abundant of these was Actinobacteria (synonym for Actinobacteriota) (22.5% mean relative abundance), followed by Proteobacteria (20.1% mean relative abundance), Acidobacteriota (11.8% mean relative abundance*),* Chloroflexi (8.8% mean relative abundance), and the archaeal phylum Crenarchaeota (7.5% mean relative abundance). In an analysis of the prokaryotic composition of the soil communities atthe Class level (Table S2), taxa of possible ecological importance could be identified in the dominant fraction of the microbiome. These included the ammonia-oxidizing archaeon Nitrososphaeria (7.4% mean relative abundance) and the photoautotrophic classes Chloroflexia (3.6% mean relative abundance) and Cyanobacteria (2.1% mean relative abundance). In addition, fourteen rare phyla (i.e., representing less than 1% of ASVs across samples) were also found in more than 50% of the samples and are therefore well represented across sub-Saharan African soils. These include the predatory bacterial phylum Bdellovibrionota, previously classified as members of the class Oligoflexia [62], members of which have been proposed to be biocontrol agents in marine environments [63], and Fribrobacteres, a phylum that includes several cellulose-degrading genera [64]. Another rare phylum identified in 589 (of 810) samples, Eremiobacterota (previously known as WS-2), includes several members capable of anoxygenic phototrophy [65] and has been recently associated with the ability to use trace gases such as hydrogen and carbon monoxide as energy sources in exothermic reactions capable of sustaining life in extreme environments [66, 67].

In an analysis of lower eukaryote diversity, eight of the 19 classified fungal phyla were identified as the dominant fraction of sub-Saharan Africa soil fungal populations, with Ascomycota (62% mean relative abundance) and Basidiomycota (14.3% mean relative abundance) being the most abundant and widespread across samples (Fig. 3). These results are consistent with previous studies showing the prevalence of these phyla in other soil microbiomes [68, 69]. The plant-symbiont mycorrhizal phylum Glomeromycota [70] was widespread across all samples, with the class Glomeromycetes (5% mean relative abundance) being found in 642 soils across all sampled countries.

### Nations exhibit distinct microbial biodiversity and community structures

To assess whether the observed differences in soil composition between countries would result in distinct soil microbiomes, both biodiversity (alpha-diversity) and community structure (beta-diversity) of the soil samples were measured and compared.

We freely acknowledge that national boundaries are artificial anthropogenic constructs and not defined by ecological zones. Nevertheless, while the use of national boundaries as an explanatory factor for the differences in continental soil microbiome structure is ecologically inappropriate, it is highly relevant in terms of the rapidly developing concepts of biodiversity (including soil microbial diversity) as a national genetic resource [71, 72].

**Fig. 4:**
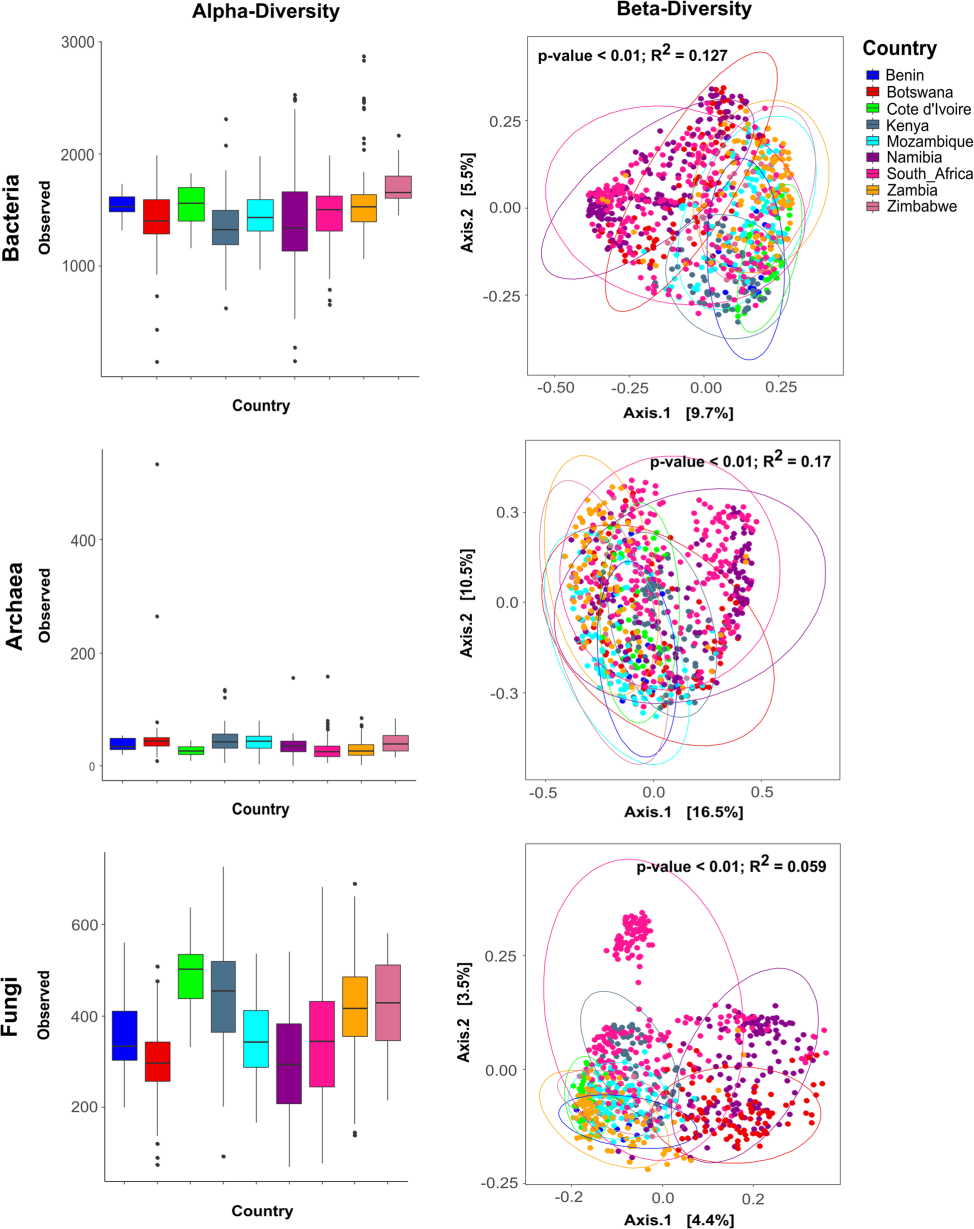
Alpha-diversity and beta-diversity of microbial communities according to country of origin. Alpha-diversity was calculated as observed number of species per sample and visualized using box-plots for the different fractions of the community (bacteria/archaea/fungi). Beta-diversity was calculated using the Bray–Curtis index and visualized as principal component analysis (PCoA) ordination plots. The different groups are highlighted by ellipses showing a 95% confidence range for the variation within each group. For both boxplots and ordination plots, samples were colored according to country of origin

Analysis of sample alpha-diversity revealed that “national” microbial communities exhibited significantly (*p*-value < 0.01) different levels of richness as indicated by the number of observed species (Fig. 4). Zimbabwe exhibited the highest bacterial biodiversity, while Cote d’Ivoire and Mozambique exhibited the highest mean number of fungal and archaeal species, respectively. These three countries exhibit a high density of forested areas (Figure S1) as well as high MAP (Figure S1). Conversely, Namibian samples exhibited the lowest number of bacterial and fungal taxa, indicating that microbial communities in Namibian soils contained a lower biodiversity relative to other sub-Saharan African countries. We suggest that this reflects the generally high aridity index and low nutrient status of most Namibian soils [73, 74].

To further explore the differences in microbial community structures between samples, beta-diversity values were calculated using the Bray–Curtis index, and the resulting scores were presented as PCoA plots and compared using the PERMANOVA test of significance (Fig. 4). Clustering of samples based on the national origin significantly explained (adjusted *p*-value < 0.01) the highest percentage of variation in community structure for the archaeal fraction of the population (*R*^2^ = 0.17), followed by the bacterial (*R*^2^ = 0.127) and fungal (*R*^2^ = 0.059) fractions. These results suggest that different sub-Saharan African nations harbor distinct soil microbial communities. However, it is important to note that the percentage of variation in microbial community structure attributed to the nation of origin is relatively small, with more than 80% of the unexplained variance (and in the case of fungal communities, more than 95%). This compositional difference between soil microbiomes of different countries could also be observed in the distribution of the dominant phyla (Figure S3). Of note is the significant (*p*-value < 0.01) over-representation of Crenarchaeota in Botswana soils, while both Cyanobacteria and Chloroflexi were over-represented in Namibia and Zambia. By comparison, Mozambique soils contained the highest percentage of Glomeromycota, while South African and Zimbabwe soils showed a significant over-representation of unknown and therefore potentially novel, fungal taxa.

### Soil chemistry and climate drive sub-Saharan Africa soil microbiomes

**Fig. 5:**
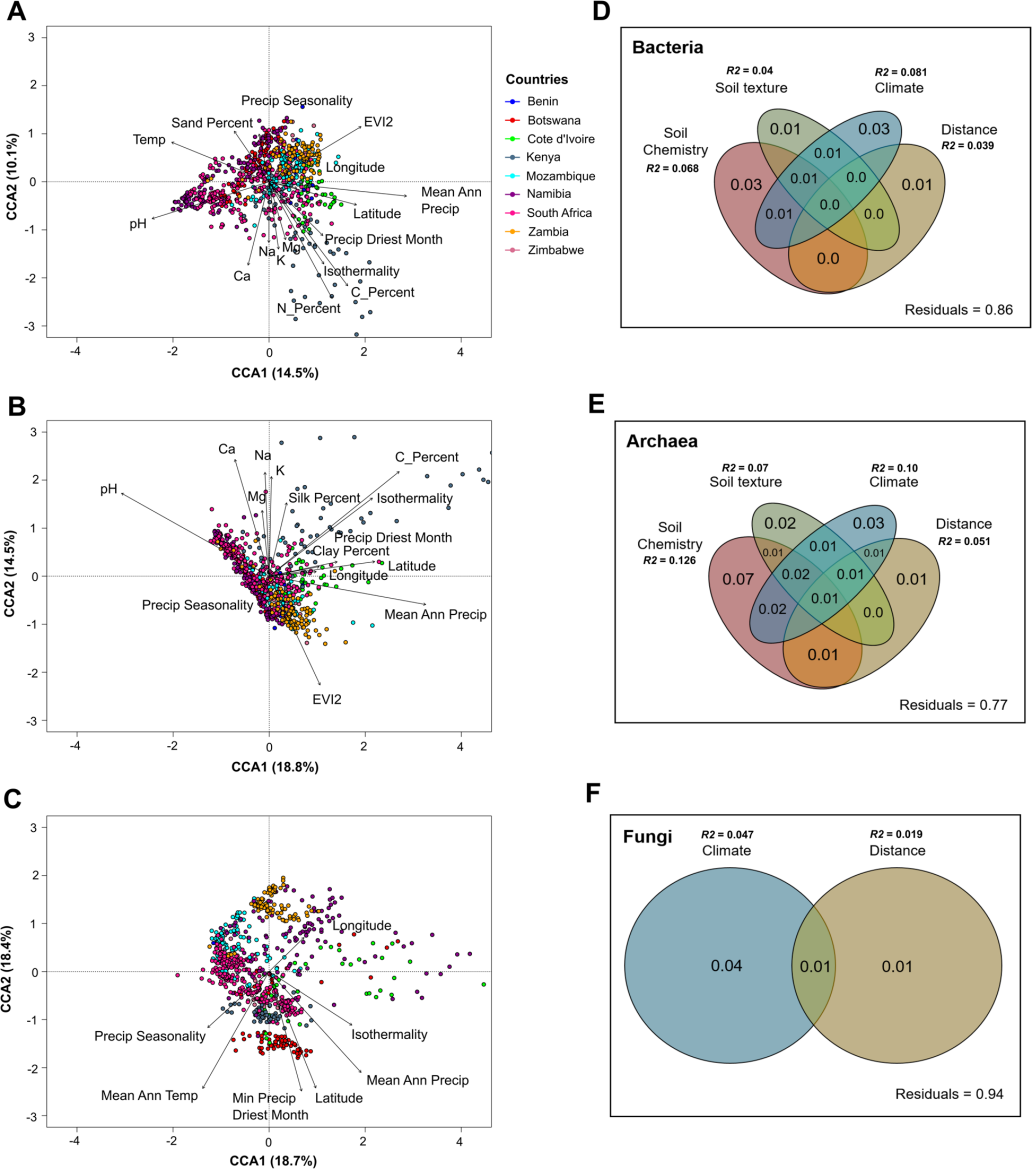
**A**–**C** Canonical correspondence analysis (CCA) plots showing the effect of explanatory climatic and chemical variables on the different fractions of the sub-Saharan Africa soil microbiome (bacteria (**A**)/archaea (**B**)/fungi (**C**)), using a significance threshold of 0.01. Percentage explained by environmental variables is expressed in the CCA1 and CCA2 axes. Samples on the plots were color-coded according to country of origin. The description and units for each variable code can be found in Table S8. **D**–**F** Venn diagrams showing the percentage of bacterial (**D**), archaeal (**E**), and fungal (**F**) community distribution explained by distinct groups of environmental variables, either individually or in combination. These percentages are expressed as a fraction between 0 and 1. The total percentage of explanatory power for each variable group (total *R*.^2^) is also indicated next to the label for each group

As noted in other landscape-scale soil microbiome studies [8, 75, 76], observed biogeographical differences in microbial biodiversity and community composition are likely to be driven by a combination of environmental factors, including soil physicochemistry and both macro- and micro-climatic factors. To assess the potential environmental drivers of the community structure of the sub-Saharan Africa soil microbiomes, a stepwise model building approach for constrained ordination models was used. The results, plotted on canonical correspondence analysis (CCA) ordination plots (Fig. 5A, B), showed that bacterial and archaeal community structures were significantly affected by several variables (adj. *p*-value < 0.01), namely pH, nutrient and cation concentration, spatial distance, vegetation cover, MAP, and precipitation seasonality. Surprisingly, nitrogen content was not predicted to be a driver of archaeal community structure, despite previous work having identified nitrogen as an important factor in shaping archaeal soil communities [77–80]. This result could be explained by the fact that the taxon in the archaeal population to be most affected by nitrogen content (i.e., Nitrososphaeria) was identified as a ubiquitous fraction of the archaeal population across the sample set. By comparison, the fungal community structure was driven by spatial scale, MAP, and MAT, as well as temperature and precipitation seasonality (Fig. 5C). As spore-formers, fungi are known for their ability to readily disperse across large distances [81, 82], and previous studies have also shown spatial scale to be a major driver of fungal community structure [83–85]. Variation partition analysis was performed to further elucidate the contribution of individual groups of explanatory variables on microbial community distribution across sub-Saharan Africa. For the prokaryotic fraction of the microbial population (Figs. 5D, E), soil chemistry was estimated to be the biggest driver of community structure, explaining 7% and 12% of the bacterial and archaeal distributions, respectively. Climate (represented by the explanatory climatic variables calculated in the CCA analysis) was estimated to be the second most important driver, explaining 8% and 10% of the distribution for both Bacteria and Archaea, respectively. Climate was also estimated to be the primary driver of fungal community structure (explaining 5% of community distribution) (Fig. 5F), with distance playing a comparatively minor role. This is again consistent with the idea that Fungi are more ubiquitously distributed due to their ability to sporulate and transverse large distances [81, 82].

Analyses of the interactions between environmental variables and the sub-Saharan African soil microbiomes can be interpreted in terms of the potential vulnerability of soil microbiome compositions to the effects of climate change. The observed importance of climatic variables such as precipitation and temperature seasonality in driving the structure of soil microbial communities suggests that changes in rainfall patterns and increased regional temperatures may have a significant impact on the microbiomes of sub-Saharan Africa. In addition, the trickle-down effects of climate change to soil chemical properties, including changes in soil pH [86] and vegetation cover [87], are likely to amplify this impact. However, it is also important to highlight the fact that a large percentage of the community structure, particularly the fungal fraction, could not be explained by the environmental factors measured in this study. It is therefore highly probable that other deterministic and stochastic factors not accounted for in this study, such as dispersal mechanisms and niche speciation [19, 21], play very important role in the microbial makeup of sub-Saharan Africa soils.

### Soil pH drives the abundance and functionality of the dominant community in sub-Saharan Africa soils

Dominant soil microbial taxa have been shown to play important roles in the ecosystem services that microbial communities provide to the surrounding environment [88–90]. Therefore, to further understand how the environment might impact the functional potential of sub-Saharan Africa soil microbiomes, random forest modeling was used to identify the environmental factors that drive the abundance of the dominant phylotypes in the community. For the purposes of this analysis, dominant phylotypes were defined according to criteria established by Delgado-Baquerizo et al. (2018b) [23]; i.e., the 10% most abundant taxa that are present in more than 50% of the sampled soils. A total of 201 bacterial, 43 fungal and 7 archaeal phylotypes were identified as dominant (Table S3), accounting for 3.1%, 1.4%, and 2.7% of the total number of phylotypes for the respective fractions of their communities. Despite this low number, the dominant phylotypes represented the majority of sequenced reads (Figure S4), a result that is consistent with the previous observation that a small fraction of the total taxa dominated sub-Saharan Africa microbial communities. It is worth noting that 134 of the dominant phylotypes were classified as rhizosphere- or root-associated, based on the manual curation of the taxonomy (Table S3) suggesting that a high percentage of taxa (53%) in dominant fraction of the community might play a role in plant-growth and soil productivity. Of the 251 dominant phylotypes, 159 bacterial (79% of dominant bacterial phylotypes), 5 fungal (12% of total dominant fungal phylotypes), and 6 archaeal (85% of dominant archaeal) phylotypes could be significantly (*p*-value < 0.05) correlated with environmental factors. These results suggest that the dominant bacterial and archaeal fractions of the sub-Saharan Africa soil microbiome are significantly impacted by the environment.

Semipartial correlation analysis was used to cluster dominant phylotypes according to the environmental factor with which they demonstrated the strongest correlation; i.e., their best environmental predictor. This analysis (Figure S5, Table S4) showed that the majority of dominant phylotypes (84 phylotypes) were significantly correlated with pH, with 51 being positively correlated, i.e., suggesting a preference for more alkaline soils, and 33 being negatively correlated, i.e., suggesting a preference for more acidic soils. A further 28 and 12 phylotypes were significantly correlated with phosphate and sodium concentrations, respectively. These results suggest that while soil microbial biodiversity and structure are driven by a combination of both climatic and soil physicochemical factors, as elsewhere [23, 24], soil pH exerts the strongest impact on the diversification and speciation of soil microbial communities in sub-Saharan Africa soils.

**Fig. 6:**
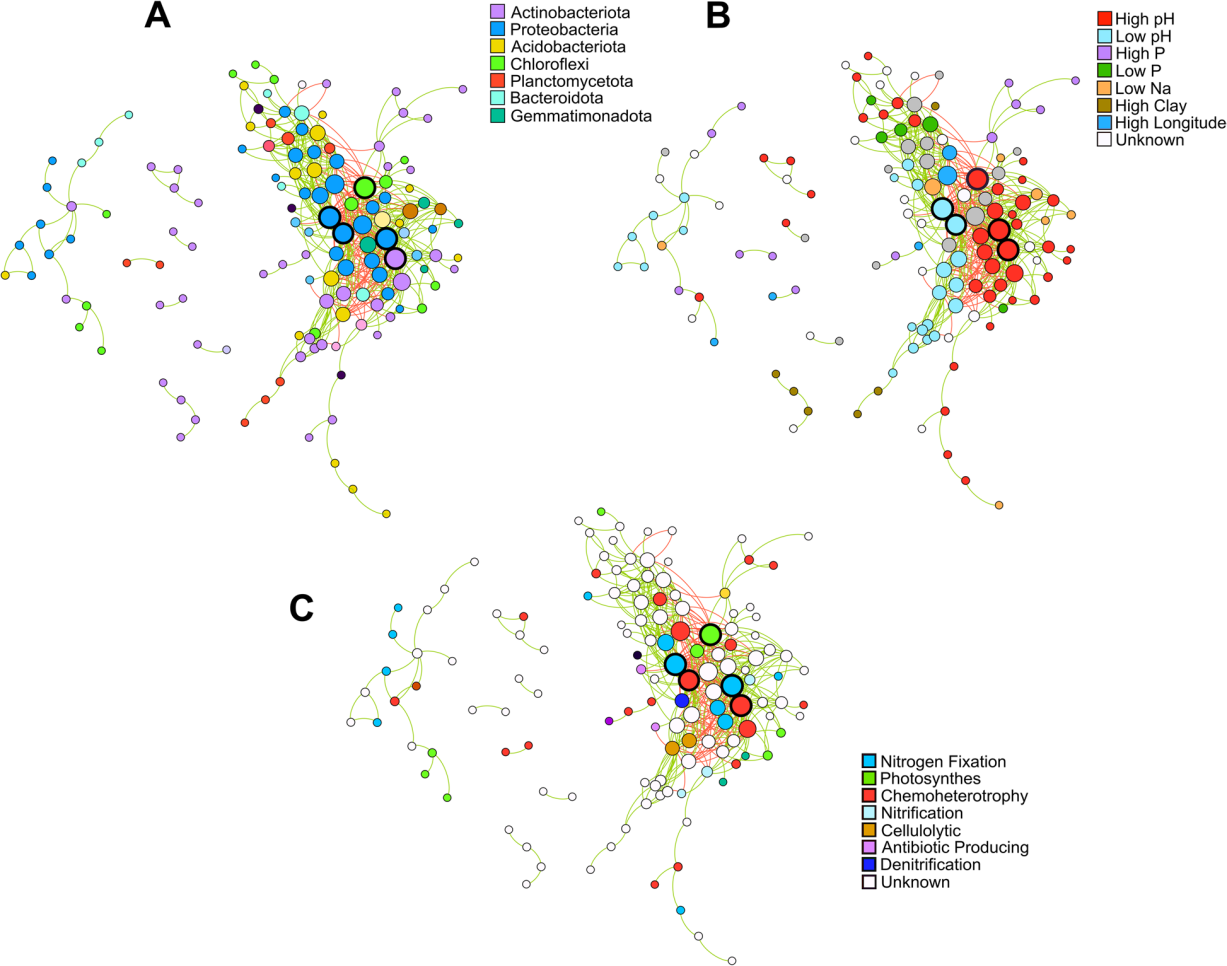
Spearman co-occurrence networks of dominant phylotypes, colored according to taxonomy (at phylum level) (**A**), associated environmental factor (based on the semipartial correlation analysis) (**B**), and function (according to FAPROTAX predictions and manual annotations) (**C**). Nodes are sized according to the number of connections (edges). Edges are colored according to nature of the correlation between nodes using the following color scheme: green—positive correlation; red—negative correlation. The top five nodes with the highest number of edges are highlighted by nodes with the thicker black perimeters

Network analysis was performed on the dominant phylotypes and combined with the FAPROTAX and manually curated functional predictions to infer the possible trophic and functional relationships between dominant phylotypes (Fig. 6, Table S3). FAPROTAX is a predictive tool that assigns a metabolic or ecological function to prokaryotic taxa based on a manually constructed database of previously functionally annotated prokaryotic clades [91]. By design, this tool is limited to prokaryotic clades that have been previously documented and is not able to account for functional traits that have not been elucidated in the literature or multiple traits within the same taxa that might not be well documented. It is therefore worth noting that information obtained from FAPROTAX were only be used to draw suggestive interpretations of the function of the dominant phylotypes, rather than conclusive information on the functional potential of the dominant community [91].

The dominant-phylotype community network was characterized by a primary cluster divided into two mutually exclusive sub-clusters of taxa (as indicated by the number of negative connections between them) that favor either high or low soil pH, supporting the conclusion that pH is a primary driver of microbial community structure in sub-Saharan Africa soils. Several phylotypes within these two sub-clusters could also be mapped to a predicted ecosystem role using FAPROTAX, including three of the five phylotypes with the highest number of connections. These include two nitrogen fixing taxa from the family *Beijerinckiaceae* and genus *Bradyrhizobium*, as well as a photosynthetic phylotype from the class Chloroflexia. These phylotypes represent potential community hubs due to their high number of connections, and their predicted functional profiles suggest that nitrogen fixation and phototrophy might be keystone functions driving the trophic relationships within the dominant fraction of sub-Saharan Africa soil microbial communities. In addition, the location of these phylotypes within the two pH-associated sub-clusters suggests that soil pH might also have a strong impact in shaping these trophic relationships in sub-Saharan Africa.

### The predicted the impact of climate change on microbial soil health

One of the primary aims of this study was to infer the potential impacts of climate change on the microbial biodiversity and composition of sub-Saharan African soil microbiomes. While microbial biodiversity alone is not a reliable measure of soil “health” [49], several studies have documented a positive correlation between soil biodiversity and soil functional redundancy and resilience [47, 50, 92, 93]. Additionally, the presence of specific rhizosphere-associated taxa, which promote ecosystem services such as nutrient cycling, has also been positively associated with plant health [50, 94, 95]. Therefore, in order to investigate the effects of climate on the viability of sub-Saharan Africa soils in terms of their microbial diversity and plant-growth-promoting potential, structural equation modeling (SEM) was used to model the causal relationships between climatic variables, soil chemistry, and microbial-biodiversity and abundance of plant-beneficial taxa. SEM is a powerful statistical tool that has been employed to test complex ecosystem models of causal interactions between abiotic and biotic factors [96–98].

**Fig. 7:**
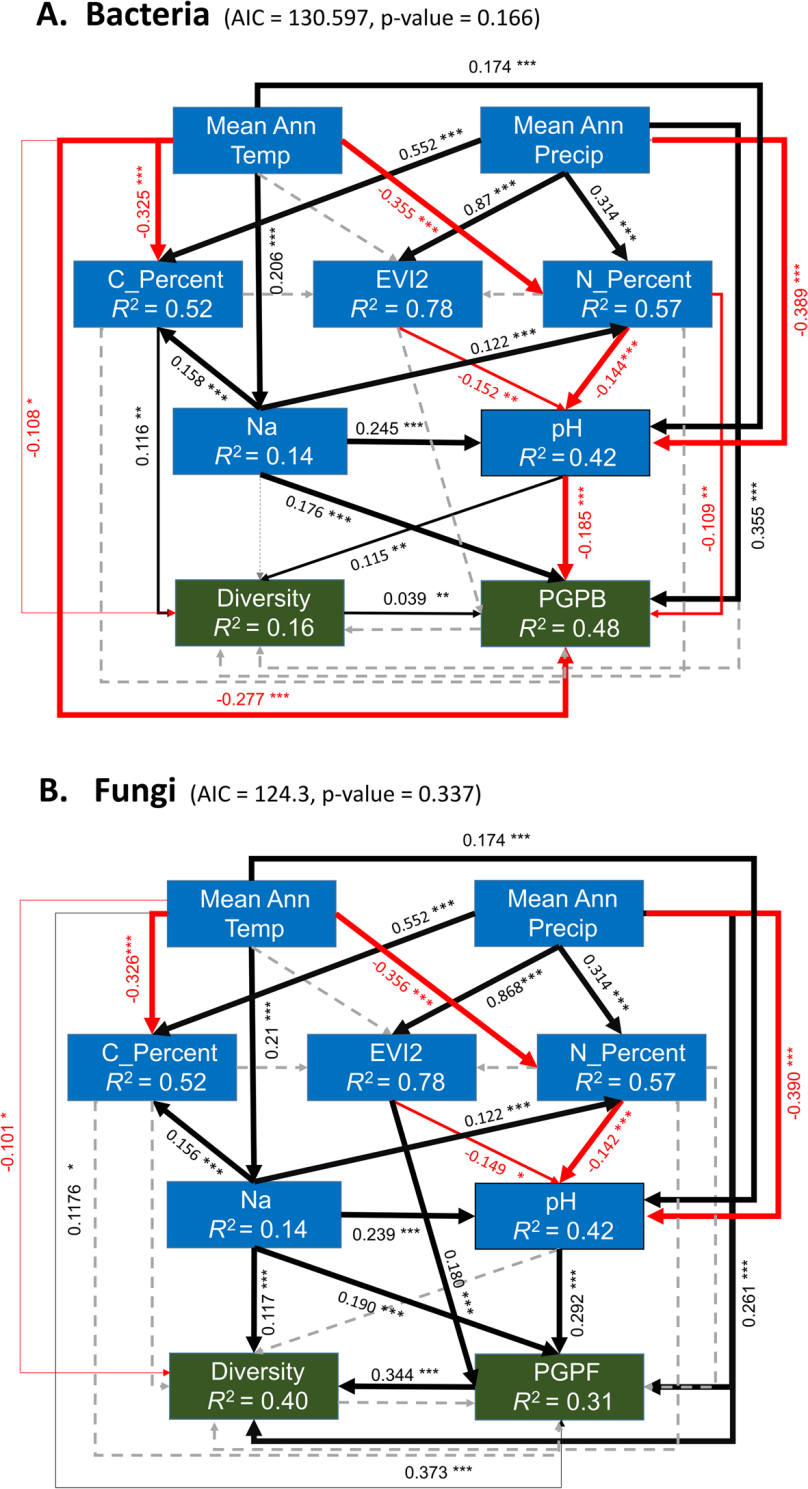
SEM models fitted to the diversity and abundance of plant-promoting taxa for bacteria (**A**) and fungi (**B**). The AIC fit metric and model *p* value are included in the top of each model. The arrows between the abiotic and biotic variables tested represent the direction and nature of the interaction between variables: black arrows represent positive interactions, while red arrows represent negative interactions. The size of the arrow signifies the significance level, with thicker arrows having higher significance (*—*p* value ≤ 0.05; **—*p* value ≤ 0.01; ***—*p* value ≤ 0.001). Gray dashed lines represent non-significant interactions from the a-priori SEM model. Standardized coefficients, representing the magnitude of the effect between variables, are also included for each interaction. The response variables used in the models are represented by dark green rectangles, while both the endogenous and exogenous variables are represented by dark blue rectangles. The fraction of explained variation (*R*.^*2*^) for each endogenous variable is highlighted below the variable

In this study, the Shannon biodiversity index and the relative abundance of rhizosphere-associated taxa with reported plant growth-promoting (PGP) capabilities (Table S5) were used as response variables. In the a priori model (Figure S6), the mean annual precipitation (MAP) was hypothesized to have a positive effect on soil nutrient content and vegetation, which in turn will have a positive effect on the microbial soil health. By comparison, the mean annual temperature (MAT) was hypothesized to have the opposite effect on biological productivity, by decreasing the vegetation density and nutrient stocks, as well as by increasing soil salt concentration and pH. The results from the SEM analysis (Fig. 7, Table S6) demonstrated that MAP was the major climatic variable positively driving the relative abundance of plant-growth-promoting bacteria (PGPB) (net std.coef. = 0.42), either through direct effect (std.coef. = 0.309) or through the positive effect on vegetation and nitrogen content, which in turn drive the acidification of soils and result in an increase in PGPB relative abundance (Fig. 7A). Several plant-promoting rhizobia have been characterized as tolerant to acidic soils [99, 100], while other PGPBs such as *Gluconacetobacter_diazotrophicus* and *Azospirillum brasilience* have been shown to grow at pH ranges between 5 and 6 [101, 102]. MAT was estimated to decrease biodiversity and PGPB abundance through direct association and through the indirect increase in soil pH (net std.coef. =  − 0.27) and decrease of soil carbon content (net std.coef. =  − 0.03). These results are consistent with previous studies showing climatic variables as major drivers of soil community structure [36,44 45, 68]. By contrast, MAP did not affect bacterial diversity directly, but rather through its indirect effects on the soil chemistry (pH, carbon, and nitrogen content) of sub-Saharan African soils (std.coef. =  − 0.045). The net negative effect of MAP on bacterial diversity is particularly difficult to explain, considering the documented positive impact of precipitation/wetting events on the microbial diversity of soils [66, 103]. However, it is worth noting that this negative impact is represented by both positive and negative pathways that might more accurately represent the complexity of the ecosystem and the interactions of abiotic factors within it. For instance, MAP is strongly associated with soil nutrient content and vegetation, which in turn were estimated to have both a positive direct and negative indirect effect on diversity through the increase in soil carbon content (std.coef = 0.098) and decrease in soil pH (std.coef =  − 0.157). Both soil total organic carbon and pH have been shown to be positively associated with bacterial biodiversity [23, 25], and the SEM tested in this study highlights the possible unimodal relationship between MAP and bacterial biodiversity, where both very high and very low precipitation inputs will have a detrimental effect on bacterial diversity. As proposed in the a priori hypothesis (Figure S6), the abundance of PGPB was positively associated with bacterial biodiversity, suggesting that more biodiverse soils harbor a greater abundance of plant-growth-promoting taxa, which might in turn have a beneficial effect on plant health in those soils.

MAP was also estimated to be the climatic factor with the strongest impact on the biodiversity and abundance of plant-growth-promoting fungi (PGPF), either by direct interaction (std.coef = 0.36 for biodiversity; std.coef = 0.30 for PGPF), or indirectly by driving vegetation density, which was estimated to have a direct association with the abundance of PGPF (std.coef = 0.144). This result suggests that the presence of PGPF in sub-Saharan African soils is strongly dependent on the presence of vegetation, which is consistent with the fact that many arbuscular mycorrhizal fungi are dependent on a symbiotic relationship with plants for nutrient acquisition [104–106]. In turn, the importance of MAP as a predictor of vegetation index has been previously demonstrated for the African continent [107]. Interestingly, MAT was estimated to have a positive effect on both fungal diversity (std.coef = 0.07) and the abundance of PGPF (std.coef = 0.149), which seems counterintuitive considering the previously reported negative impact of temperature on plant cover and soil aridity [108, 109]. However, the annual mean temperature of the sites sampled in this study (ranging from 12.4 to 28.9 °C) corresponds to a temperature range where fungal taxa are likely to show optimal activities and growth rates [110]. The fact that MAT has no significant effect on EVI2 in the predicted SEM also suggests that in sub-Saharan Africa, precipitation might be a more important predictor than the temperature for vegetation cover. Another result worth noting is the estimated positive association of Na to both the abundance of PGPT as well as fungal diversity, which is also counter-intuitive considering the documented detrimental effect of salt on microbial diversity and root-associated taxa [111, 112]. However, as for the MAT data, the Na concentrations measured for the sampled dataset (ranging from 0 to 1 g/kg of soil) fall below the estimated inhibitory concentrations of NaCl for soil microbial communities [113], and these results suggest that at low concentrations Na might exert a beneficial effect on soil microbial communities.

In contrast to the bacterial model, pH was not significantly associated with fungal diversity and was instead estimated to have a positive effect on the abundance of PGPF, which in turn was positively associated with fungal diversity. While increases in pH have been previously shown to have detrimental effects on fungal diversity [109], arbuscular mycorrhizal fungi are characterized by a preference for neutral and alkaline environments [114, 115], which is reflected in the SEM model. More importantly, the SEM predicted a direct positive association between the abundance of PGPF and fungal diversity, suggesting that arbuscular mycorrhizal fungi are not only key players in maintaining soil health and plant growth [50, 116], but might also be important in maintaining fungal biodiversity.

To further assess the impact of climate change on sub-Saharan Africa soil biological productivity, SEM modeling was used to estimate the changes in biodiversity and abundance of plant-promoting microbiota in 2050 and 2100, as a response to predicted changes in MAT and MAP driven by an increase in carbon emissions. The MIROC6 climate model, which is widely employed for future climate predictions [117, 118], was used to predict MAT and MAP values for 2040–2060 and 2080–2100 temporal windows, under two different shared socio-economic pathway (SSP) scenarios for greenhouse (GH) emissions (SSP1-2.6 and SSP5-8.5) (Figure S7). Under this model, the mean annual temperatures are predicted to rise for all sub-Saharan African countries sampled in this study, with an estimated mean rise of 4 °C by the 2090s under the worst GH emission scenario (SSP5-8.5). By comparison, predicted precipitation profiles are more regional, with countries such as Kenya and Mozambique showing a predicted decrease in MAP, while Benin and Zambia are predicted to have higher MAP indexes by 2100. Both climate scenarios modeled in MIROC6 were predicted to have a significant (*p*-value < 0.01) negative effect on the prokaryotic productivity of sub-Saharan African soils (Fig. 8S A-B), with the biggest impact predicted for the 2080–2100 window under the highest GH emission scenario. The prokaryotic communities in Kenyan soils were estimated to be the most vulnerable to the predicted climate change, with a noticeable reduction in biodiversity (average 0.68% reduction in Shannon index) and abundance of PGPB (average 41% reduction in relative abundance values) being predicted for the 2040–2060 period under the SSP1-2.6 scenario. In countries such as Benin, South Africa, and Zambia, a comparable reduction in biodiversity was only recorded for the most extreme scenario (average 0.53%, 0.54%, and 0.78% reduction in Shannon index, respectively), suggesting that the soil prokaryotic communities in these countries will be more resilient to climate change. In contrast, soil fungal diversity and the abundance of plant-growth-promoting fungi were predicted to either remain constant or increase in six of the sub-Saharan countries sampled in this study (with the exception of Kenya, Mozambique, and Zimbabwe) (Figure S8C-D). The two countries where fungal biodiversity and abundance of PGPF were predicted to increase as early as the 2050s under the SSP1-2.6 scenario, Benin and Cote d’Ivoire, were also predicted to have increased annual precipitation by the 2050s. These results again suggest the importance of precipitation as a main climatic driver of soil fungal diversity and community structure (as shown by the CCA results), while prokaryotic communities are more vulnerable to extreme temperature shifts. These results also suggest that in future climate change scenarios, fungal communities across sub-Saharan soils will be more resilient to anthropogenic-driven climate disruptions, a conclusion that is consistent with recent research showing increased resilience of fungal communities to climate change in different parts of the world [119, 120].

## Conclusion

This study represents the most extensive survey of the sub-Saharan soil pan-microbiome performed to date and fills a substantial gap in our knowledge of the microbial ecology of sub-Saharan Africa. The results from this study largely corroborate the general global patterns reported by Fierer and others (see refs 8, 10, and 23 for examples); that is, that sub-Saharan African soils are dominated by a small but ubiquitous fraction of the microbial population and that pH is the primary driver in shaping this fraction of the soil community. As the dominant fraction of soil communities is thought to play an important role in ecosystem servicing, scenarios of disturbance such as the ones emulated here are expected to affect soil functioning and productivity. More importantly, we have demonstrated, through the constrained and structural equation modeling, the vulnerability of microbial communities in sub-Saharan Africa to climate change. The observed roles of precipitation and temperature in shaping the diversity and structure of sub-Saharan Africa soil microbiomes imply that climate change will have a significant impact on the latter. This was highlighted by the SEM models, which hinted at the complex roles of climate in modulating the chemistry and biological productivity of sub-Saharan African soils. The vulnerability of sub-Saharan African soil microbial communities to climate change was further demonstrated by the prediction of future climate change scenarios, in which the prokaryotic fraction of the soil microbiomes is predicted to be negatively impacted by shifts in temperature and precipitation patterns. Considering the important role that prokaryotes play in soil ecosystem services, and that the economies of many countries in sub-Saharan Africa are very heavily reliant on rain-fed agriculture, the predicted climate-driven decrease in soil prokaryotic diversity and abundance of plant-growth-promoting bacteria are likely to exacerbate the negative economic effects of climate change. By comparison, fungal communities were predicted to be more resilient to climate shifts, and future studies should focus on whether this resilience can mitigate the adverse effects of climate change.

This study also demonstrated that different countries harbor distinct soil microbiomes. However, as discussed above, this difference is linked to spatial-scale variation in environmental factors and soil chemistry, rather than an inherent property of each country. It is worth noting however that the different policies that individual countries enact on land use and management might have an indirect impact on shaping soil microbial communities by regulating anthropogenic impacts on soils. It is our hope that the findings from this study will assist future policy makers on the best measures to preserve and protect sub-Saharan African soil microbiomes in the face of climate change.

## Materials and methods

### Sample collection

A total of 810 sites were sampled across nine countries in sub-Saharan Africa (Table S7), between January 2017 and June 2018. The number of sites allocated to each nation was calculated on the basis of land area, with a total allocation of 1000 effective sample sites, in order to cover as much sub-continental area as possible. Sampling sites were spaced at approximately 50 km, using the major and minor road networks in each nation to maximize regional coverage. A standardized protocol was used throughout the sample collection campaign to avoid biases in the downstream analysis of the microbial community. Briefly, four 200 g surface soil (0–5-cm depth) sample replicates were collected in sterile Whirlpak® bags from the vertices of a 100 m x 50 m plot at each GPS-located sample site. Each 200 g sample was a composite of four 50 g pseudo-replicate sub-samples recovered from within a 1-m^2^ quadrat. Samples were stored on ice and transported to the country’s host institutions, where they were stored at 4 °C before being shipped to the Centre for Microbial Ecology and Genomics (CMEG) (University of Pretoria, South Africa) for nucleic acid extraction and soil physicochemical analysis. Upon arrival to CMEG, samples were sieved (4 mm mesh) to remove plant roots and other debris and stored at − 80 °C.

### DNA extraction and amplicon sequencing

Before nucleic acid extraction, the four replicate samples from each site were thoroughly mixed into a composite sample. DNA was subsequently extracted from 0.25 g of soil using the DNeasy PowerSoil Kit (QIAGEN, USA) following the manufacturer’s instructions with minor modifications. Specifically, the elution buffer C6 was pre-heated to 55 °C for 10 min before the final elution step, and the DNA was eluted using 70 μl of the elution buffer. After extraction, DNA concentration and purity were checked using the Nanodrop 2000 (ThermoFisher, USA) and agarose gel electrophoresis.

DNA samples were sent to MRDNA laboratories (www.mrdnalab.com, Shallowater, TX, USA) for sequencing of the V4/V5 16S rRNA gene and the ITS-1 and ITS-2 subunits of the internal transcribed spacers (ITS). Primers used were the 515F (5′-GTGYCAGCMGCCGCGGTAA-3′) and 909R (5′-CCCCGYCAATTCMTTTRAGT-3′) for the 16S rRNA gene amplification [121], and ITS1F (5′-CTTGGTCATTTAGAGGAAGTAA-3′) and ITS4 (5′-TCCTCCGCTTATTGATATGC-3′) for ITS amplification [122]. Before library preparation, the regions of interest were amplified using the HotStarTaq Plus Master Mix Kit (Qiagen, USA) and subsequently purified using calibrated Ampure XP beads (Beckman Coulter Life Sciences, USA). Sequencing was performed on an Illumina MiSeq instrument following the manufacturer’s guidelines.

### Sequence processing and taxonomic annotation

The raw amplicon reads were filtered, trimmed, and clustered into unique amplicon sequence variants (ASVs) using the QIIME2 pipeline [123]. Briefly, raw sequences were demultiplexed and denoised and merged using DADA2 [124] to filter chimeric sequences and singletons. The reads were further truncated at the 5′- and 3′-termini to remove sequences below a quality score of 25. The truncation lengths used were 10 bps for the 5′-termini, and 290 and 220 bps for the 3′-termini of 16S rRNA and ITS reads, respectively. Reads were subsequently clustered into ASVs and classified into taxonomic phylotypes using a trained SILVA 138 (release 12–2019) database for prokaryotic species [125] and the UNITE fungal database (release 11–2018) [126].

### Soil physicochemical analysis

Soil physicochemical properties were analyzed at the University of Pretoria, following the protocols outlined by AgriLASA (2004) [127] (Table S1). A total of eleven soil physicochemical parameters were analyzed. The soil pH was measured using the slurry method at a 1:2.5 soil/water ratio, and the pH of the supernatant was recorded with a calibrated benchtop pH meter (Crison Basic, + 20, Crison, Barcelona, Spain). The Mehlich 3 test was used to determine the concentrations (mg/kg) of soluble and exchangeable sodium (Na), potassium (K), carbon (Ca), magnesium (Mg), and phosphorus (P) [128]. The extractable ion concentration was quantified using ICP-OES (Inductively Coupled Plasma Optical Emission Spectrometry, Spectro Genesis, SPECTRO Analytical Instruments GmbH & Co. KG, Germany). The soil particle size distribution (sand/silt/clay percentage) was measured using the Bouyoucos method [129]. The total nitrogen (TN) and soil organic carbon (TOC) (as a percentage) were measured using the catalyzed high-temperature combustion method (Dumas method) [130].

### Extraction of macroclimate variables

The GPS coordinates of each sampling site were used to obtain eight bioclimatic data variables (Table S1) from WorldClim2 Global Climate Data [131] at a resolution of 30-arc seconds. These included mean annual temperature (Bio1, MAT, °C), temperature seasonality (Bio4, °C*100), minimum temperature of the coldest month (Bio6, °C*10), mean annual precipitation (Bio12, MAP, mm), temperature isothermality (Bio3, %), precipitation of the driest month (Bio14, mm), and precipitation seasonality (Bio15, %). The Enhanced Vegetation Index-2 (EVI2) was obtained from the NASA Land Processes Distributed Active Archive Center’s (LP DAAC) VIIRS Vegetation Indices dataset [132], at a 500-m resolution. Land cover categories (LC) were determined based on the ESA GlobCover 2009, 300-m resolution, 22 class, and global land cover map [133]. The 22 land cover classes of the land cover map were defined based on the United Nations (UN) Land Cover Classification System (LCCS). The ESA GlobCover 2009 land cover classification was used as it provides fair differentiation between the broad African vegetation, agricultural, and urban classes. The relevant bioclimatic variables, enhanced vegetation index, and land cover class were extracted for each soil sample point, at the native resolution of each dataset.

### Statistical analyses

#### Soil chemistry and climatic differences across sub-Saharan countries

Significant differences in soil chemistry and climatic variables between countries were calculated using the stats (version 3.6.2) package in RStudio version 4.0.3 [134]. The normality of the dataset was first tested with the Shapiro–Wilk test [135]. The Kruskal–Wallis rank-sum test [136] was subsequently used to calculate the significance of mean differences in variables between countries, and the pairwise Willcoxon rank-sum test [137] was used to compare significant differences between country groups (adj. *p*-value < 0.01). The distribution of climatic and soil chemistry variables across different sites was calculated on log-standardized data using the “prcomp” function, which performs a principal component analysis of the data (PCA) [138]. The resulting distance matrix between samples was plotted in a PCA graph, with the projected direction and magnitude of the distribution for each variable plotted in a separate loading plot. The hmisc (version 4.5) package was subsequently used to calculate strong significant Pearson correlations [129] between variables (adj. *p* value < 0.01), which were plotted in a bubble graph using the corrplot (version 0.9) package.

#### Identification of environmental drivers of microbial diversity and community structure

Metrics for biodiversity (alpha-diversity) and community structure (beta-diversity) were calculated using the vegan (version 2.5.7) [139] and phyloseq (version 1.16.2) [140] packages in Rstudio. Both observed richness and the Shannon index [141] were used as metrics for alpha-diversity. Before calculation of alpha-diversity values, the ASV tables were rarefied to a read depth of 27,000 and 11,400 total reads for prokaryotic and fungal ASVs, respectively. The prokaryotic ASV table was subsequently split into Archaea and Bacteria using the “subset_taxa” function in phyloseq. Differences in alpha-diversity between countries were assessed using the methodology described above for the climatic and soil chemistry variables.

The Bray-Cutis beta-diversity index of each sample was calculated from the log-transformed (natural log) rarefied ASV tables using the “vegdist” function in vegan. The distribution of samples according to their beta-diversity was subsequently plotted on a principal component analysis plot (PCoA) [142], and the significance of beta-diversity dissimilarity between countries was calculated using Permutational multivariate analyses of variance (PERMANOVA) [143] with 999 permutations. The environmental (climate and soil) drivers of microbial community structure were estimated using constrained correspondence analysis (CCA) [144]. The climatic/soil chemistry dataset was *z*-score standardized and tested for multicollinearity using the “vif” function from the car (version 3.0.11) package [145]. Variables with vif values above 10 were before performing the CCA. The best models for explanatory variables were calculated using the forward stepwise regression model selection method with the ordistep() function in the vegan package, with 1000 permutations. The significance of the best-fitted models and each predictor variable in the model were calculated using the ANOVA permutation test for CCA [146], with 1000 permutations. The variation partition analysis was performed on the log-transformed ASV datasets and the standardized environmental variables dataset using the varpart() function in the vegan package.

#### Identification of dominant phylotypes and their environmental preferences

In this study, phylotypes were identified at the species level, i.e., sharing ≥ 99% sequence identity across the amplified reads. Dominant phylotypes were subsequently defined according to criteria established by Delgado-Baquerizo et al. (2018b) [23], i.e., as the top 10% most abundant taxa that are present in more than 50% of the sampled soils. To identify the environmental preferences of the dominant phylotypes, random forest analysis [147] was performed on the rarefied counts for each dominant phylotype using the randomForest (version 4.6.14) package in R. The models tested with the algorithm included all the numerical environmental variables used in multivariate constrained analyses described above. A threshold of > 30% of the explained variation was used to consider phylotypes with a potential environmental preference. Semi-partial correlations (Spearman) analysis was subsequently performed using the ppcor package [148] to identify the contribution of each environmental variable in explaining the distribution of each phylotype with an environmental preference. Phylotypes were subsequently clustered into ecological groups according to the predictor with the highest correlation value, at a *p* value threshold of 0.05. The ecological groups were defined by the nature of the correlation between phylotype and the predictor, e.g., phylotypes exhibiting a negative correlation with pH would be clustered into a low pH ecological group. The relative abundance of each ecological group per sample was subsequently calculated by averaging the rarefied relative abundances of phylotypes belonging to the group in each sample.

#### Network analysis of the dominant community

To assess possible biotic relationships between dominant phylotypes, co-occurrence network analysis was performed on the rarified absolute count table of the dominant phylotypes. Pairwise Spearman correlations between all dominant phylotypes were first calculated, after which correlations below *r* < 0.65 and with non-significant *p* values (threshold *p* value < 0.00001) were filtered out. A very stringent significance threshold was chosen to prevent the high number of false positives normally associated with Spearman correlations [149]. The resulting pairwise correlation table was used to draw a co-occurrence network, which was visualized in gephi [150]. This software was also used to calculate the topology properties of the network, including degrees, betweenness centrality, and modularity. The potential functional roles of each phylotype in the network were predicted using the FAPROTAX taxonomy-based functional prediction software [91] as well as by manual curation, linking the phylotype with previous research on functionality. Nodes in the network were colored based on the taxonomy, ecological group, and predicted function of the dominant phylotype.

#### Structural equation modeling of climate effect on soil health

Structural equation modeling (SEM) [96–98] was used to model the effects of climate on the chemistry and health of sub-Saharan African soils. In this study, we used confirmatory path analysis to assess the validity of an a priori ecosystem model of causal relationships (Figure S6), which was informed by both the multivariate constrained analyses and previous literature showing a link between the various abiotic and biotic variables used in the model. The Shannon diversity indexes and relative abundances of plant-growth-promoting taxa (PGPT) were used as proxy measures for bacterial and fungal diversity as well as the abundance of taxa that might exert a positive effect on plant growth and health in the ecosystem. The environmental parameters chosen for SEM analysis were the following: MAP, MAT, TOC (carbon content), TN (nitrogen content), Na concentration (mg/kg), EVI2, and pH. These environmental variables have also been shown repeatedly to be associated with microbial community biodiversity and structure [10, 25, 109]. The package piecewiseSEM [98] in R was used to assess the a priori model and calculate the best fit model, using the author’s suggested workflow (https://github.com/jslefche/piecewiseSEM/blob/master/vignettes/piecewiseSEM.Rmd). Tables containing the Shannon indexes, the average relative abundance of PGPT, and the measurements for the environmental variables per sample were log-normalized before modeling each interaction in the a priori model using the “lme” function. The country of origin was used as a random effect correction, while latitude/longitude was used for spatial autocorrelation correction, to account for human differences in the sampling strategy. In SEM analysis, the best fitting models are considered to be those that are similar as possible to the measured data, i.e., that do not reject the null hypothesis (*p* value > 0.01). Best-fit models were therefore chosen based on the highest *p* value, lowest AIC and BIC scores, and highest parsimony. The net estimated effect of each environmental variable on the response variables was estimated by summing the effects of direct and indirect pathways. In turn, indirect pathways were calculated as the product of the effects from the connections that make up the pathway.

#### MIROC6-based predictions of the effect of climate change on the microbial biodiversity and productivity

Annual mean temperature (Bio1) and precipitation (Bio12) projections were retrieved for two future CMIP6 climatic scenarios, respectively, representing the most optimistic mitigation-driven and worst concentration-driven shared socio-economic scenarios of GH emissions (SSP1-2.6 and SSP5-8.5) [151] calculated with the model MIROC6 [117]. Spatial climate data forecasts of the plot future conditions were retrieved with UP licensed ArcMap 10.2 software [152] for two periods (2040–2960 and 2080–2100) from WoldClim2—future climate data repository (https://www.worldclim.org/data/cmip6/cmip6climate.html) at 30-arc second resolution. The predict() function from the car package (version 3.0.11) [153] in RStudio was subsequently used to calculate all the predicted values for the endogenous and response variables of the optimal SEM model, based on the forecasted climate MAT and MAP values for both climate scenarios and both year periods.

## Supplementary Information

**Additional file 1. Figure S1. **Distribution of samples across the 9 African countries according to their land cover (LC) classification. Land cover codes used were the following: LC_1 - Rainfed croplands; LC_2 - Mosaic Cropland (50-70%) / Vegetation (grassland, shrubland, forest) (20-50%); LC_3 - Mosaic Vegetation (grassland, shrubland, forest) (50-70%) / Cropland (20-50%); LC_4 - Closed to open (>15%) broadleaved evergreen and/or semi-deciduous forest (>5m); LC_5 - Closed (>40%) broadleaved deciduous forest (>5m); LC_6 - Open (15-40%) broadleaved deciduous forest (>5m); LC_10 - Mosaic Forest/Shrubland (50-70%) / Grassland (20-50%); LC_11 - Mosaic Grassland (50-70%) / Forest/Shrubland (20-50%); LC_12 - Closed to open (>15%) shrubland (<5m); LC_13 - Closed to open (>15%) grassland; LC_14 - Sparse (>15%) vegetation (woody vegetation, shrubs, grassland); LC_17 - Closed to open (>15%) vegetation (grassland, shrubland, woody vegetation) on regularly flooded or waterlogged soil; LC_18 - Artificial surfaces and associated areas (urban areas >50%); LC_19 - Bare areas. 

**Additional file 2. Figure S2. **Significant (*p*-value < 0.01) variation of soil chemistry and climatic variables across African countries. Significance was calculated using the Kruskal-Wallis test for non-parametric data distributions, while pair-wise comparison was calculated using the pairwise Wilcox test. Significant results are indicated using the following nomenclature: * - *p*-value < 0.05; ** - *p*-value < 0.01; *** - *p*-value < 0.001.  

**Additional file 3.Figure S3. **Average relative abundance of the top bacterial (A) and fungal (B) taxa across the sampled sub-Saharan Africa countries.

**Additional file 4.Figure S4. **Relative abundance of dominant (201 bacterial, 43 Fungal and 7 archaeal) phylotypes across soil samples. 

**Additional file 5.** **Figure S5.** Relationship between the relative abundance of dominant phylotypes across soil samples and their main environmental predictors, as determined by semipartial correlation analysis. Phylotypes were grouped into environmental categories based on the correlation between phylotype and its major environmental predictor: positive correlation with pH – high pH; negative correlation with pH – low pH; positive correlation with phosphate – high Phosphate; negative correlation with phosphate – low Phosphate; negative correlation with Sodium – low Sodium.

**Additional file 6.** **Figure S6.** A-priori ecological model tested using SEM. MAP and MAP are represented as exogenous variables (black rectangles), soil chemistry and vegetation index are represented as endogenous variables (blue rectangles), while the Shannon diversity and abundance of PGPT are represented as response variables (green rectangles). The color and direction of the arrows represent the nature and direction of the causal relationships between variables: red – negative relationship; black – positive relationship.

**Additional file 7. Figure S7. **MIROC6 model predictions for mean annual temperature (oC) (A) and mean annual precipitation (mm) (B) under too different GH emission scenarios (SSP126 and SSP585), predicted for 2040-2060 and 2080-2100 temporal windows. The predicted datasets are grouped according to country, as indicated by the vertical dashed lines. 

**Additional file 8. Figure S8-A.** Predicted prokaryotic Shannon biodiversity index values (expressed as natural log scale) in soils of the 9 sub-Saharan Africa countries used in this study, for 2040-2060 and 2080-2100 under two distinct GH emission scenarios (SSP126 and SSP585), and comparison with current predicted Shannon biodiversity as estimated by SEM. Pairwise significance values of differences in biodiversity means between the different years and scenarios are represented by the brackets with the following nomenclature: * - *p*-value < 0.05; ** - *p*-value < 0.01; *** - *p*-value < 0.001.  

**Additional file 9. Figure S8-B. **Predicted abundance values of PGPB (expressed as natural log scale) in soils of the 9 sub-Saharan Africa countries used in this study, for 2040-2060 and 2080-2100 under two distinct GH emission scenarios (SSP126 and SSP585), and comparison with current predicted Shannon biodiversity as estimated by SEM. Pairwise significance values of differences in biodiversity means between the different years and scenarios are represented by the brackets with the following nomenclature: * - *p*-value < 0.05; ** - *p*-value < 0.01; *** - *p*-value < 0.001.

**Additional file 10. Figure S8-C. **Predicted fungal Shannon biodiversity  values (expressed as natural log scale) in soils of the 9 sub-Saharan Africa countries used in this study, for 2040-2060 and 2080-2100 under two distinct GH emission scenarios (SSP126 and SSP585), and comparison with current predicted Shannon biodiversity as estimated by SEM. Pairwise significance values of differences in biodiversity means between the different years and scenarios are represented by the brackets with the following nomenclature: * - *p*-value < 0.05; ** - *p*-value < 0.01; *** - *p*-value < 0.001.

**Additional file 11. Figure S8-D. **Predicted abundance values of PGPF (expressed as natural log scale) in soils of the 9 sub-Saharan Africa countries used in this study, for 2040-2060 and 2080-2100 under two distinct GH emission scenarios (SSP126 and SSP585), and comparison with current predicted Shannon biodiversity as estimated by SEM. Pairwise significance values of differences in biodiversity means between the different years and scenarios are represented by the brackets with the following nomenclature: * - *p*-value < 0.05; ** - *p*-value < 0.01; *** - *p*-value < 0.001.

**Additional file 12. Table S1. **Metadata for all the sites used in the study, which include the latitude and longitude GPS coordinates, physicochemical properties of the sample soils, macroclimatic variables for each site, and soil texture and land cover classifications based on the macroclimatic variables.

**Additional file 13. Table S2. **Taxonomy of prokaryotic taxa in the dominant fraction of the microbial community, at the Class taxrank. 

**Additional file 14. Table S3. **Metadata of the dominant phylotypes, including taxonomy, functional predictions (based on FAPROTAX and manual curation), and ecological groups based on the main environmental predictor.

**Additional file 15. Table S4. **Table with the semi-partial correlation analysis results, in which the correlation values (r) and associated p-values of the variable with the highest correlative value are displayed for each dominant phylotype that was significantly (p-value < 0.05) correlated with environmental factors. 

**Additional file 16. Table S5. **Taxonomy of the taxa considered as plant-growth-promoting. 

**Additional file 17. Table S6. **Net estimates and corresponding significance values for the environmental variables associated with soil health in the SEM model.

**Additional file 18. Table S7. **Number of samples allocated for each country, and number of samples collected. 

**Additional file 19. Table S8. **Variable codes, meaning and units for the environmental variables used in this study. 

## Data Availability

The sequencing data analyzed in this study has been deposited in the SRA NCBI submission portal (BioProject ID PRJNA807934). The R scripts used for the analysis of the sequencing data can be found in the GitHub page https://github.com/PedroHLebre/AfSM_scripts.
