# Supplementary figures and images for: Biogeographical survey of soil microbiomes across sub-Saharan Africa: structure, drivers, and predicted climate-driven changes
Source: Microbiome. 2022 Aug 23;10:131. doi: 10.1186/s40168-022-01297-w (PMC9396824; doi:10.1186/s40168-022-01297-w)

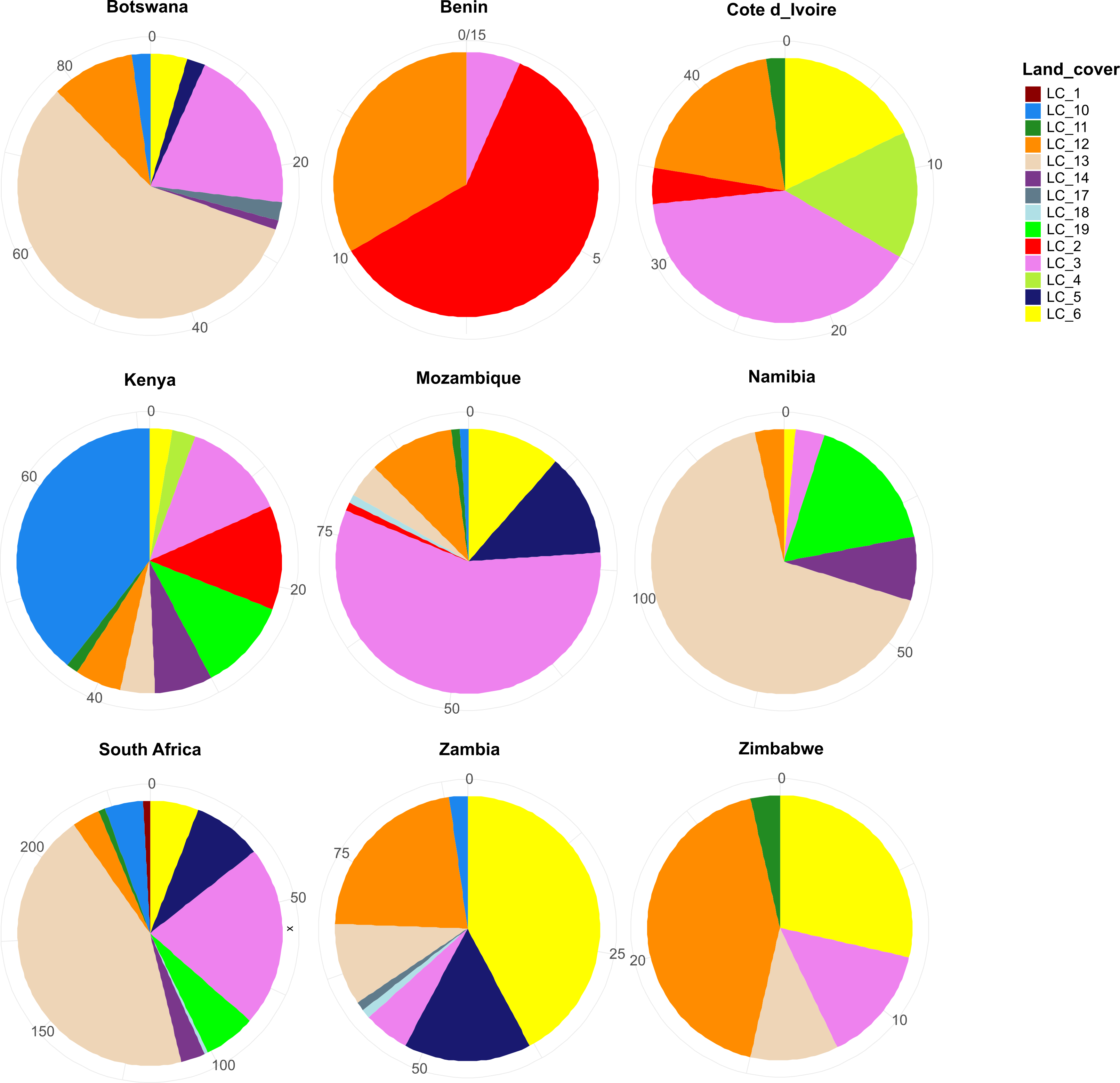

Supplement: Supplementary file 2 — Additional file 1. Figure S1. Distribution of samples across the 9 African countries according to their land cover (LC) classification. Land cover codes used were the following: LC_1 - Rainfed croplands; LC_2 - Mosaic Cropland (50-70%) / Vegetation (grassland, shrubland, forest) (20-50%); LC_3 - Mosaic Vegetation (grassland, shrubland, forest) (50-70%) / Cropland (20-50%); LC_4 - Closed to open (>15%) broadleaved evergreen and/or semi-deciduous forest (>5m); LC_5 - Closed (>40%) broadleaved deciduous forest (>5m); LC_6 - Open (15-40%) broadleaved deciduous forest (>5m); LC_10 - Mosaic Forest/Shrubland (50-70%) / Grassland (20-50%); LC_11 - Mosaic Grassland (50-70%) / Forest/Shrubland (20-50%); LC_12 - Closed to open (>15%) shrubland (<5m); LC_13 - Closed to open (>15%) grassland; LC_14 - Sparse (>15%) vegetation (woody vegetation, shrubs, grassland); LC_17 - Closed to open (>15%) vegetation (grassland, shrubland, woody vegetation) on regularly flooded or waterlogged soil; LC_18 - Artificial surfaces and associated areas (urban areas >50%); LC_19 - Bare areas. [file 40168_2022_1297_MOESM1_ESM.tiff]

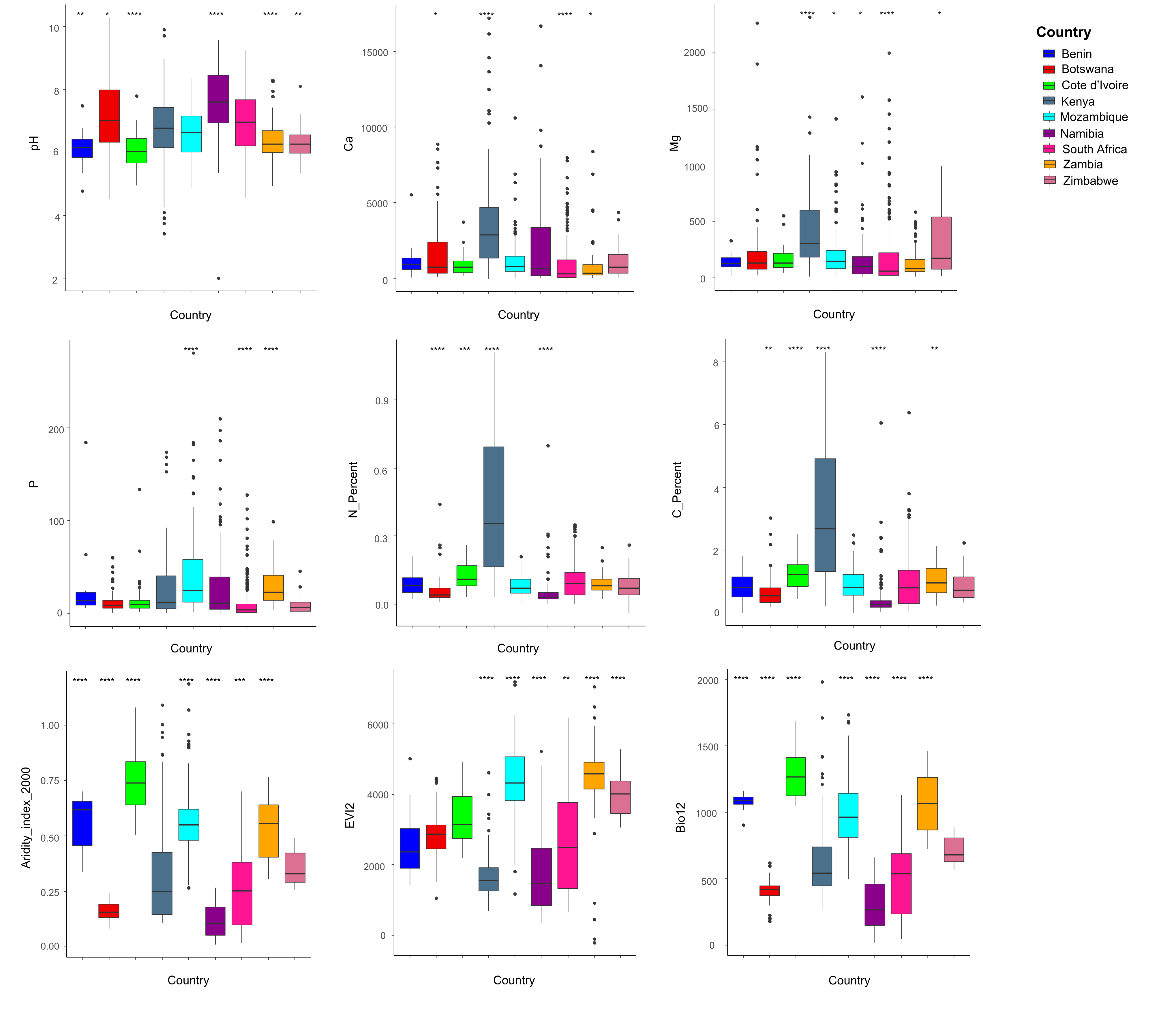

Supplement: Supplementary file 3 — Additional file 2. Figure S2. Significant (p-value < 0.01) variation of soil chemistry and climatic variables across African countries. Significance was calculated using the Kruskal-Wallis test for non-parametric data distributions, while pair-wise comparison was calculated using the pairwise Wilcox test. Significant results are indicated using the following nomenclature: * - p-value < 0.05; ** - p-value < 0.01; *** - p-value < 0.001. [file 40168_2022_1297_MOESM2_ESM.tiff]

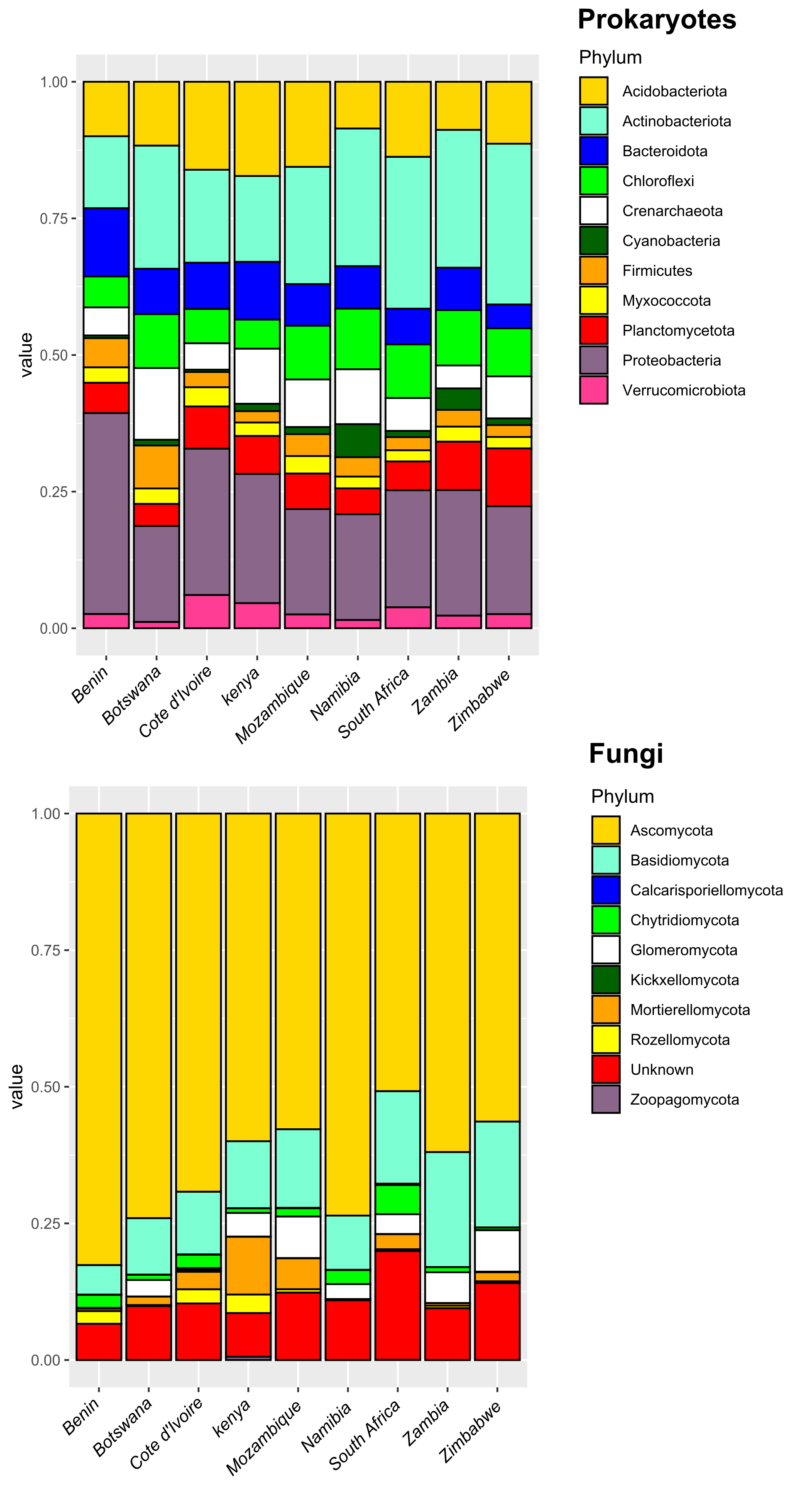

Supplement: Supplementary file 4 — Additional file 3.Figure S3. Average relative abundance of the top bacterial (A) and fungal (B) taxa across the sampled sub-Saharan Africa countries. [file 40168_2022_1297_MOESM3_ESM.tiff]

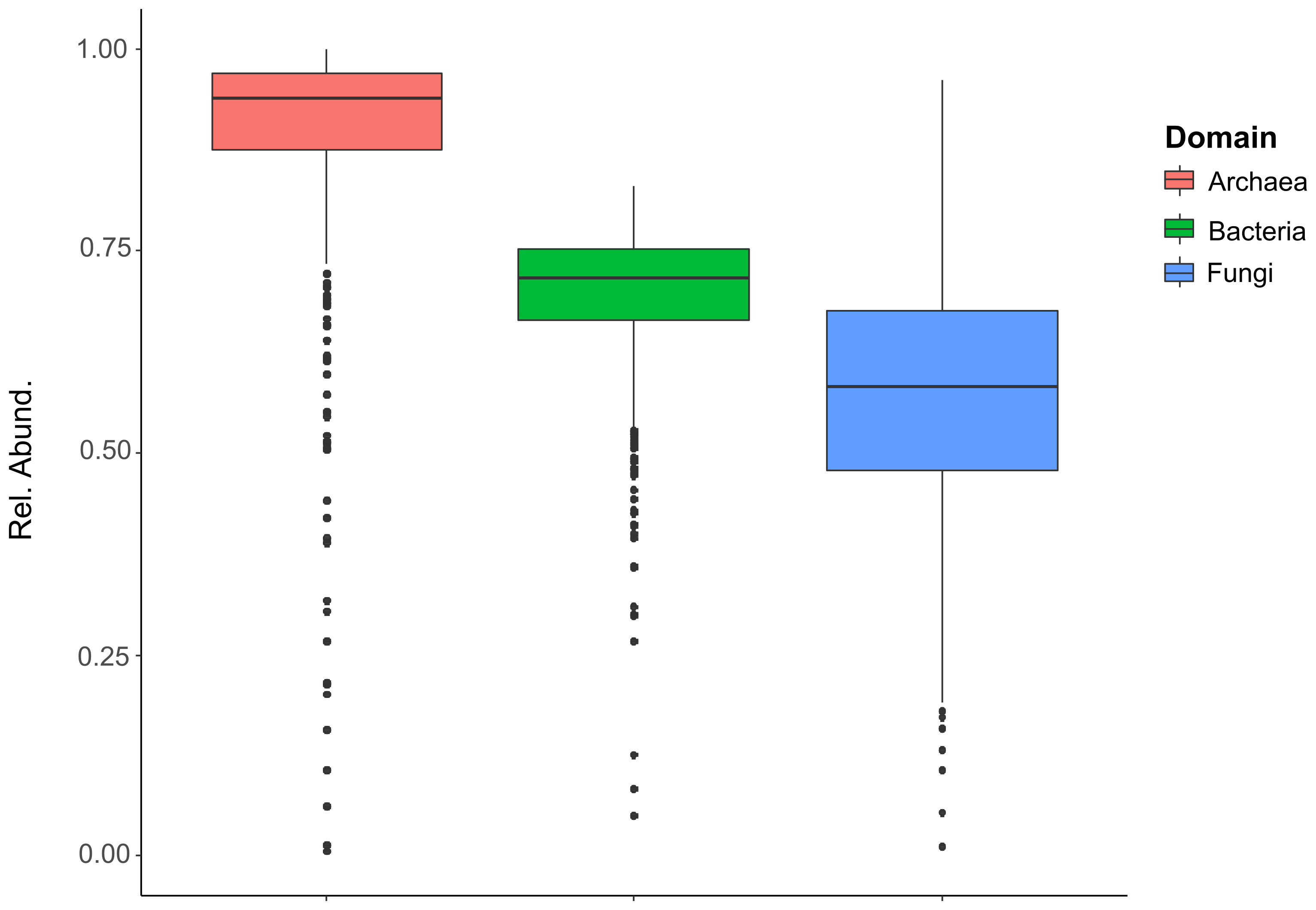

Supplement: Supplementary file 5 — Additional file 4.Figure S4. Relative abundance of dominant (201 bacterial, 43 Fungal and 7 archaeal) phylotypes across soil samples. [file 40168_2022_1297_MOESM4_ESM.tiff]

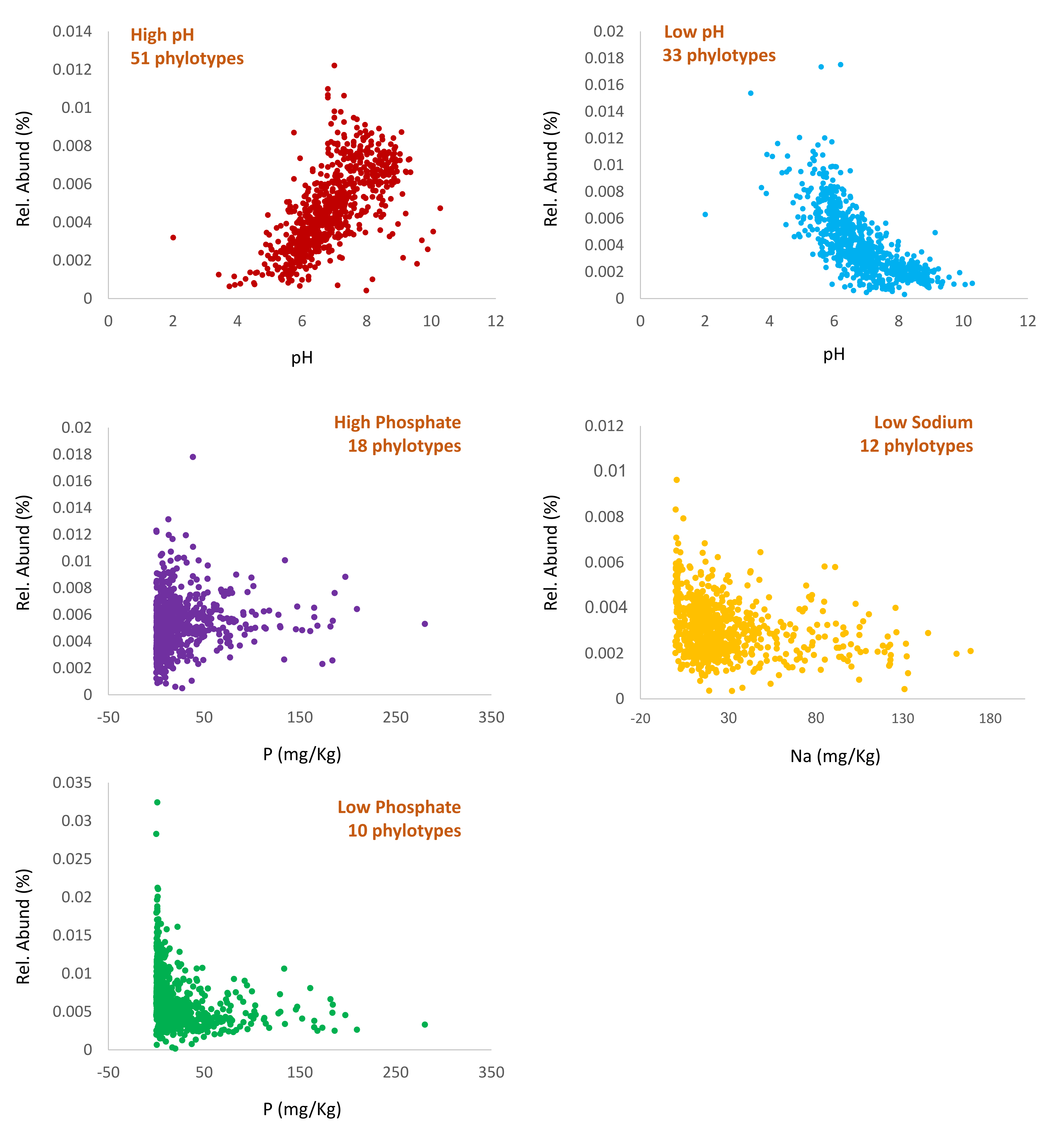

Supplement: Supplementary file 6 — Additional file 5. Figure S5. Relationship between the relative abundance of dominant phylotypes across soil samples and their main environmental predictors, as determined by semipartial correlation analysis. Phylotypes were grouped into environmental categories based on the correlation between phylotype and its major environmental predictor: positive correlation with pH – high pH; negative correlation with pH – low pH; positive correlation with phosphate – high Phosphate; negative correlation with phosphate – low Phosphate; negative correlation with Sodium – low Sodium. [file 40168_2022_1297_MOESM5_ESM.tiff]

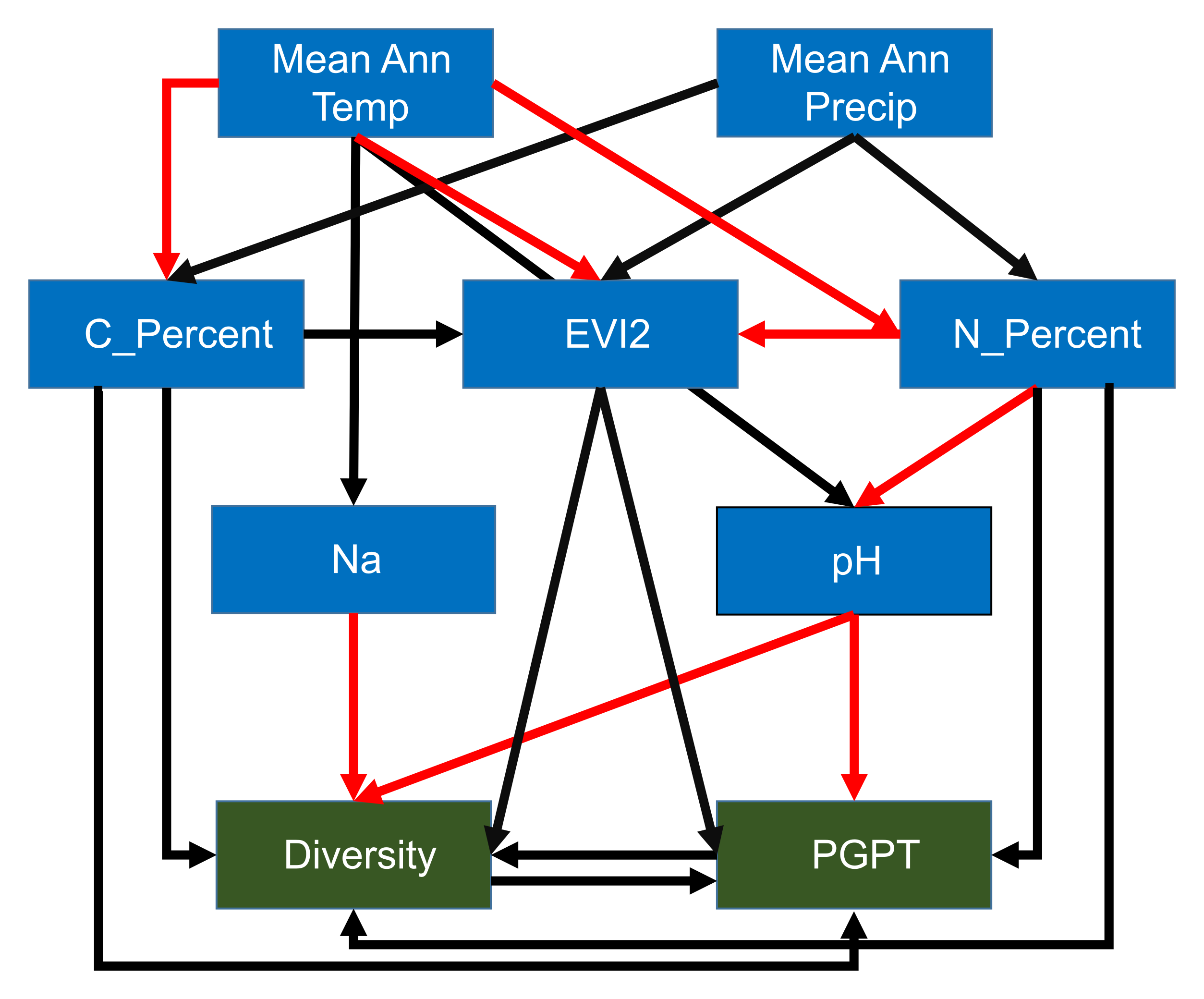

Supplement: Supplementary file 7 — Additional file 6. Figure S6. A-priori ecological model tested using SEM. MAP and MAP are represented as exogenous variables (black rectangles), soil chemistry and vegetation index are represented as endogenous variables (blue rectangles), while the Shannon diversity and abundance of PGPT are represented as response variables (green rectangles). The color and direction of the arrows represent the nature and direction of the causal relationships between variables: red – negative relationship; black – positive relationship. [file 40168_2022_1297_MOESM6_ESM.tiff]

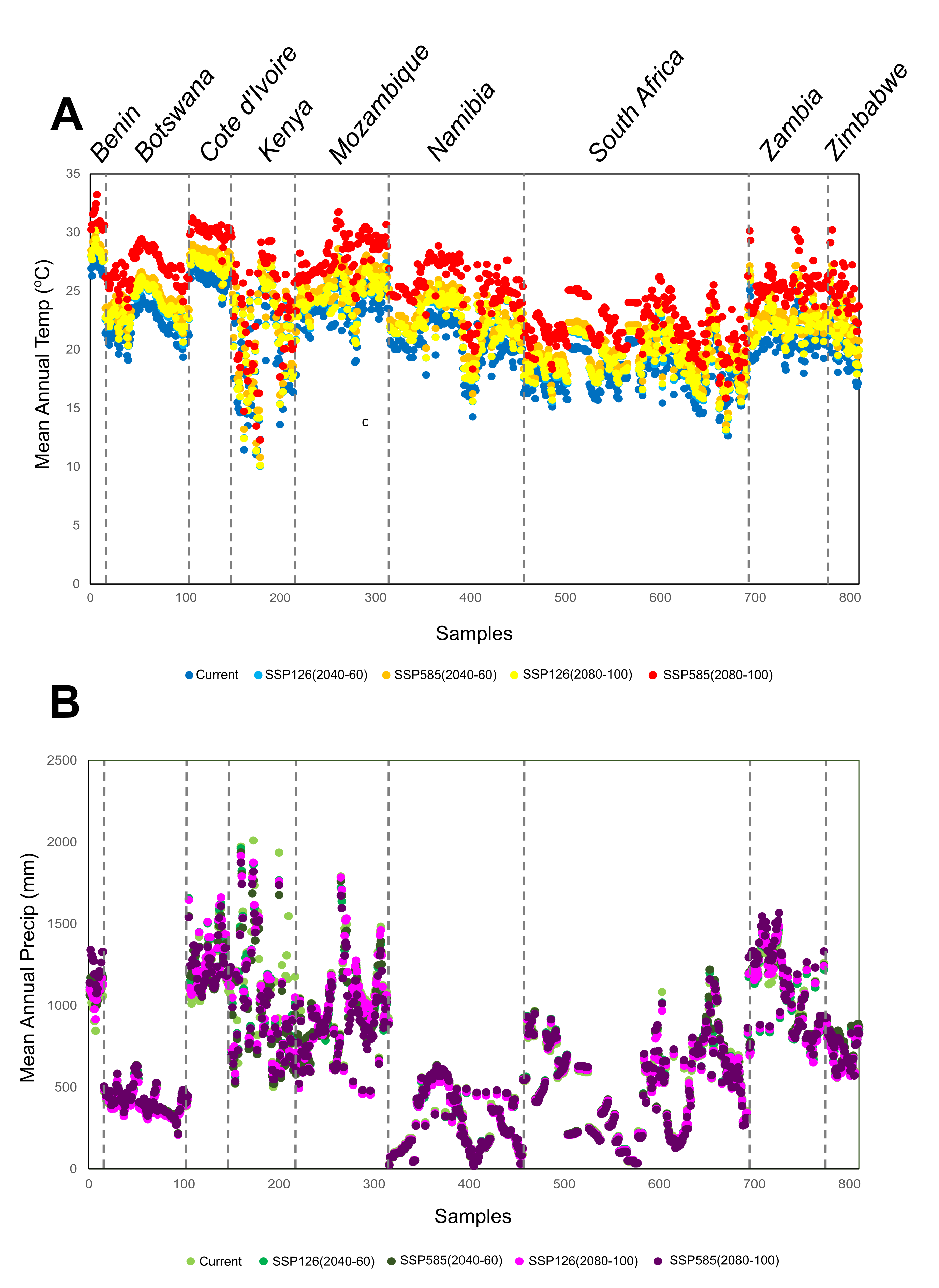

Supplement: Supplementary file 8 — Additional file 7. Figure S7. MIROC6 model predictions for mean annual temperature (oC) (A) and mean annual precipitation (mm) (B) under too different GH emission scenarios (SSP126 and SSP585), predicted for 2040-2060 and 2080-2100 temporal windows. The predicted datasets are grouped according to country, as indicated by the vertical dashed lines. [file 40168_2022_1297_MOESM7_ESM.tiff]

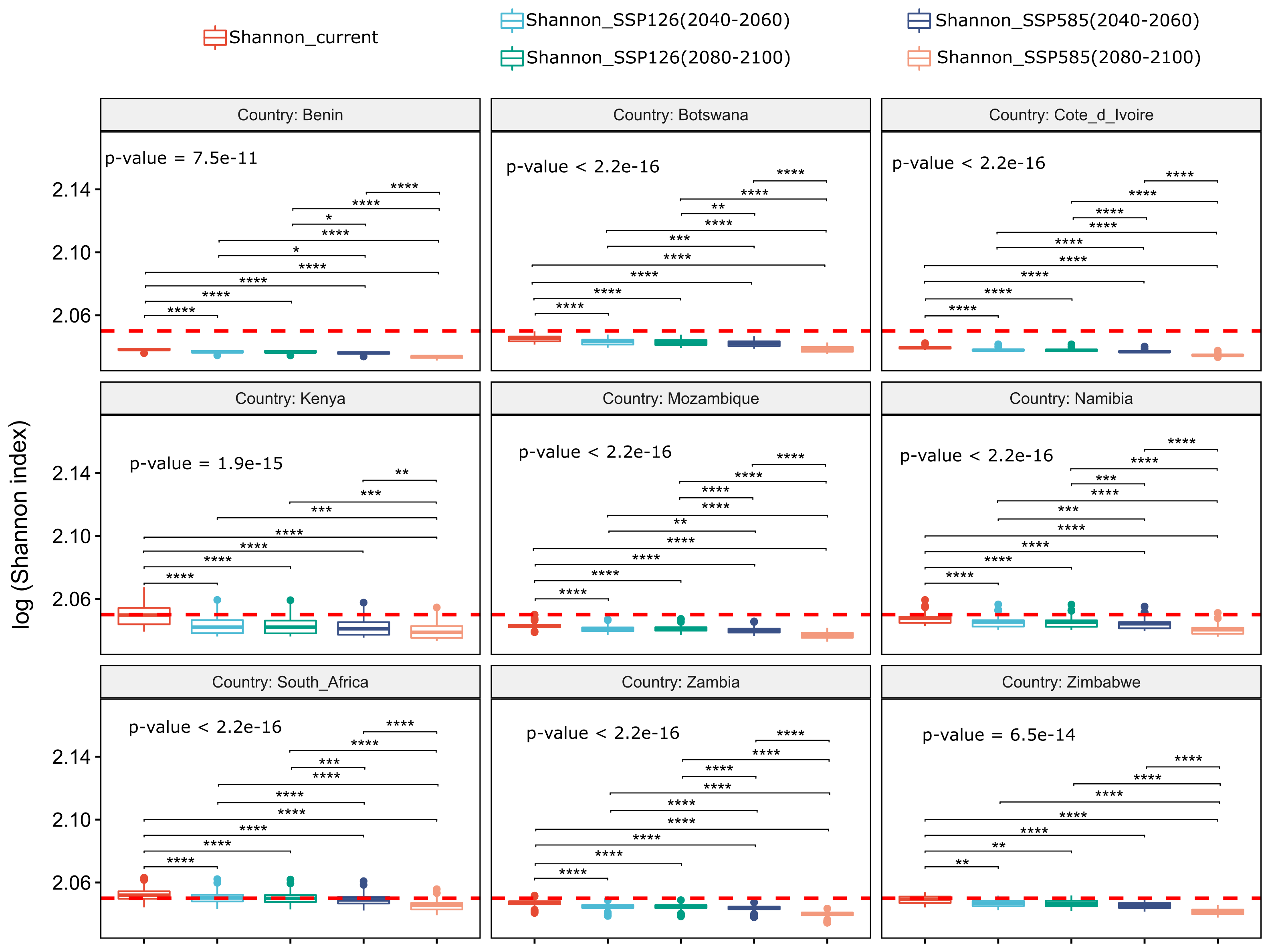

Supplement: Supplementary file 9 — Additional file 8. Figure S8-A. Predicted prokaryotic Shannon biodiversity index values (expressed as natural log scale) in soils of the 9 sub-Saharan Africa countries used in this study, for 2040-2060 and 2080-2100 under two distinct GH emission scenarios (SSP126 and SSP585), and comparison with current predicted Shannon biodiversity as estimated by SEM. Pairwise significance values of differences in biodiversity means between the different years and scenarios are represented by the brackets with the following nomenclature: * - p-value < 0.05; ** - p-value < 0.01; *** - p-value < 0.001. [file 40168_2022_1297_MOESM8_ESM.tiff]

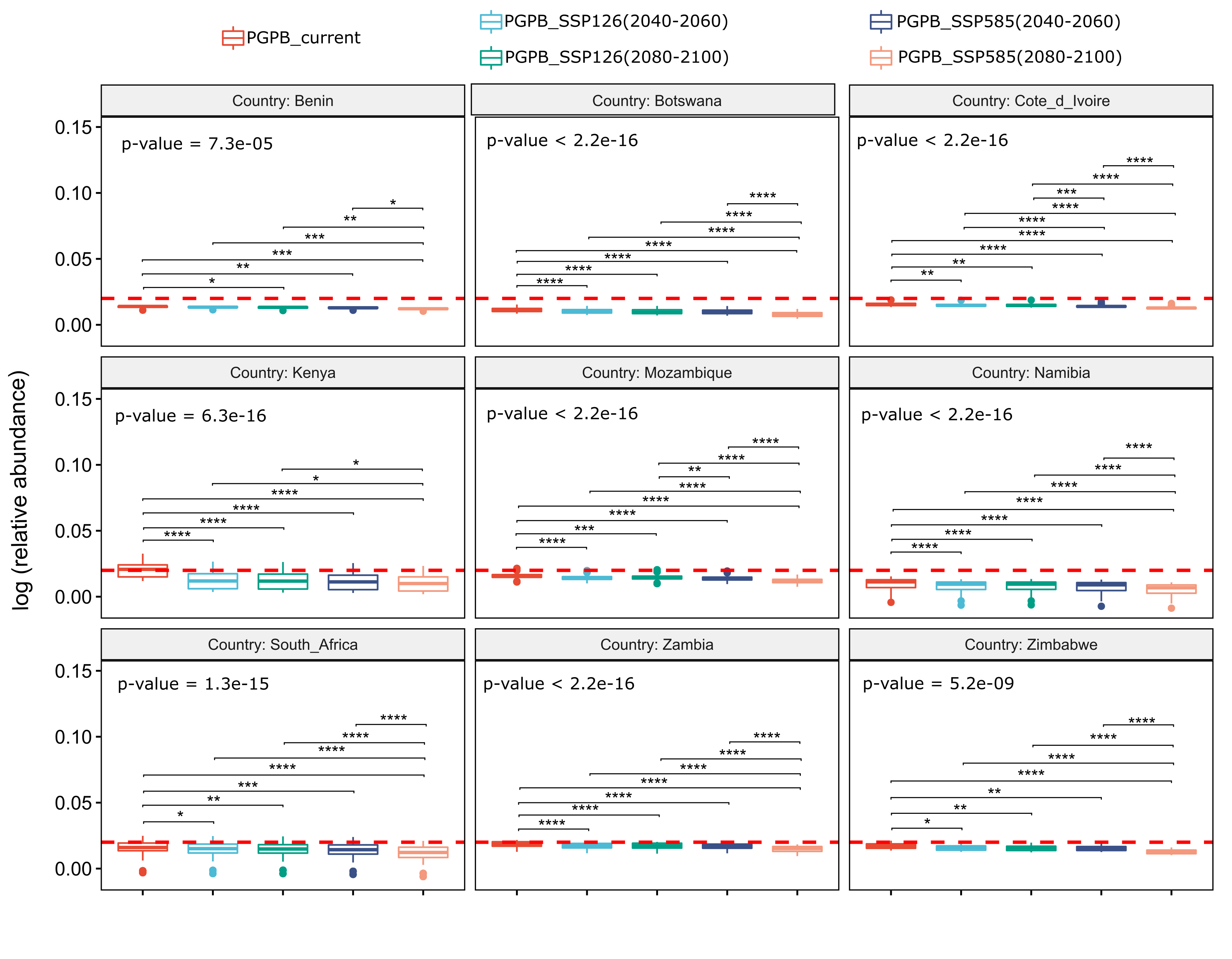

Supplement: Supplementary file 10 — Additional file 9. Figure S8-B. Predicted abundance values of PGPB (expressed as natural log scale) in soils of the 9 sub-Saharan Africa countries used in this study, for 2040-2060 and 2080-2100 under two distinct GH emission scenarios (SSP126 and SSP585), and comparison with current predicted Shannon biodiversity as estimated by SEM. Pairwise significance values of differences in biodiversity means between the different years and scenarios are represented by the brackets with the following nomenclature: * - p-value < 0.05; ** - p-value < 0.01; *** - p-value < 0.001. [file 40168_2022_1297_MOESM9_ESM.tiff]

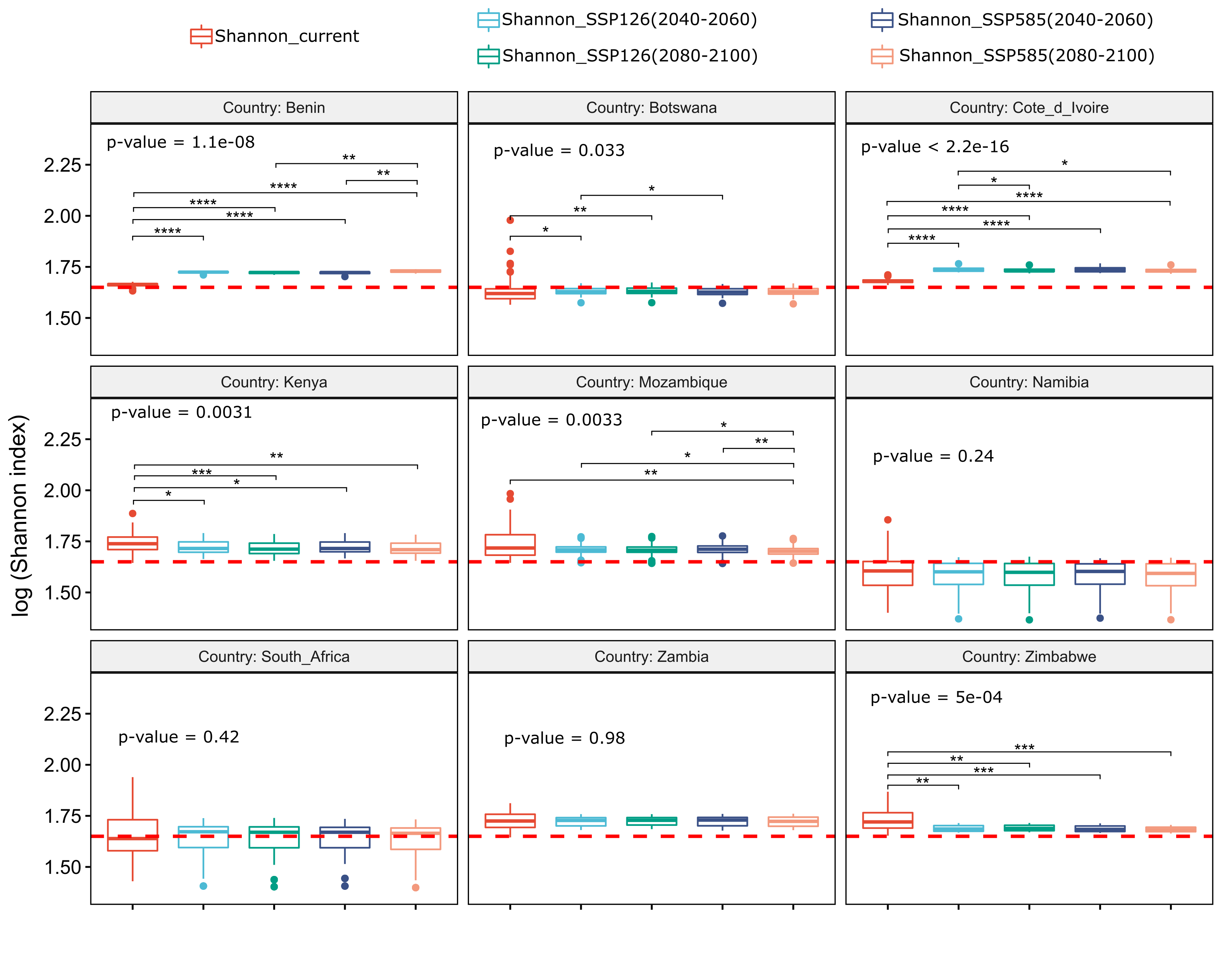

Supplement: Supplementary file 11 — Additional file 10. Figure S8-C. Predicted fungal Shannon biodiversity values (expressed as natural log scale) in soils of the 9 sub-Saharan Africa countries used in this study, for 2040-2060 and 2080-2100 under two distinct GH emission scenarios (SSP126 and SSP585), and comparison with current predicted Shannon biodiversity as estimated by SEM. Pairwise significance values of differences in biodiversity means between the different years and scenarios are represented by the brackets with the following nomenclature: * - p-value < 0.05; ** - p-value < 0.01; *** - p-value < 0.001. [file 40168_2022_1297_MOESM10_ESM.tiff]

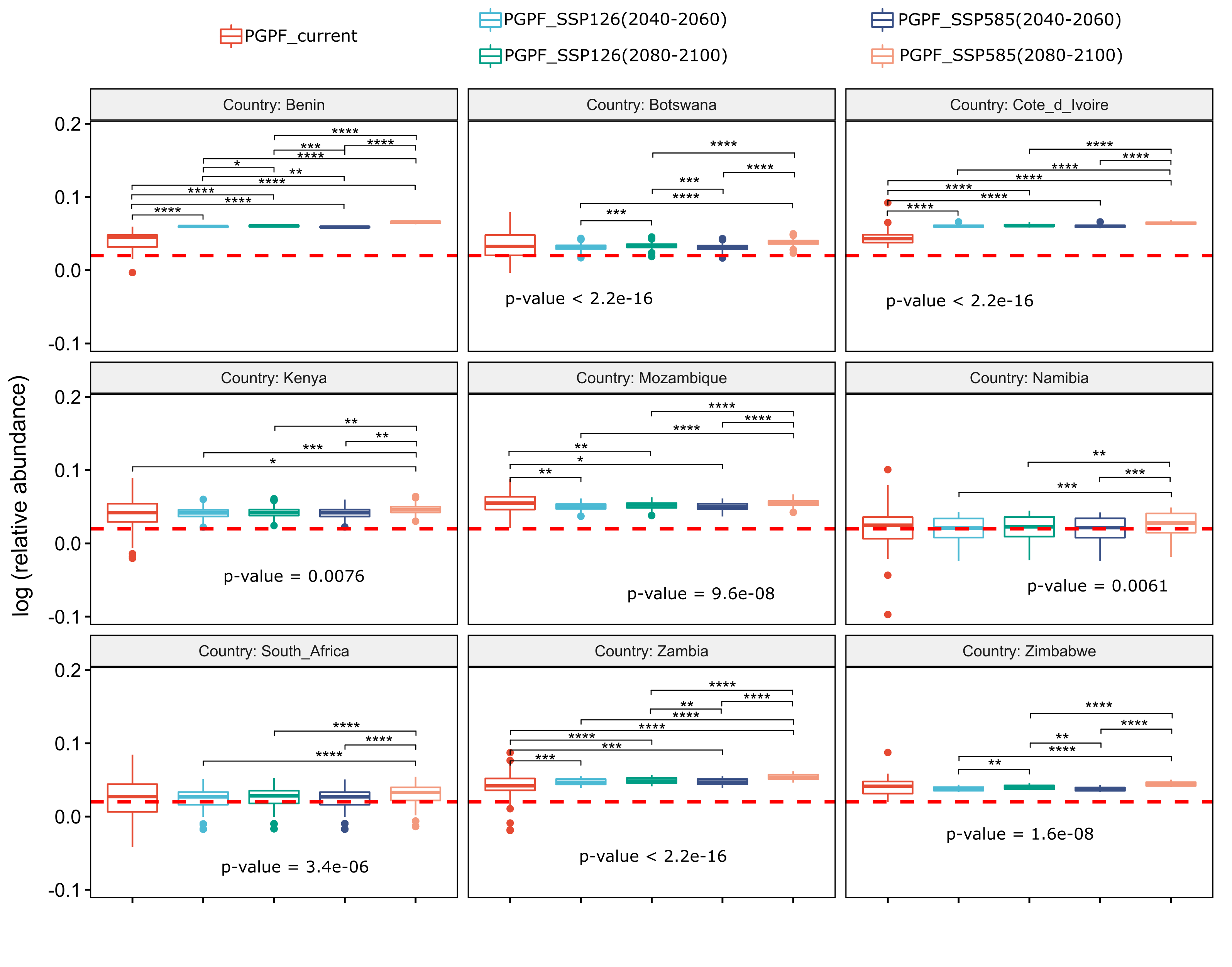

Supplement: Supplementary file 12 — Additional file 11. Figure S8-D. Predicted abundance values of PGPF (expressed as natural log scale) in soils of the 9 sub-Saharan Africa countries used in this study, for 2040-2060 and 2080-2100 under two distinct GH emission scenarios (SSP126 and SSP585), and comparison with current predicted Shannon biodiversity as estimated by SEM. Pairwise significance values of differences in biodiversity means between the different years and scenarios are represented by the brackets with the following nomenclature: * - p-value < 0.05; ** - p-value < 0.01; *** - p-value < 0.001. [file 40168_2022_1297_MOESM11_ESM.tiff]
